# Mutations of PI3K-AKT-mTOR pathway as predictors for immune cell infiltration and immunotherapy efficacy in dMMR/MSI-H gastric adenocarcinoma

**DOI:** 10.1186/s12916-022-02327-y

**Published:** 2022-04-21

**Authors:** Zhenghang Wang, Xinyu Wang, Yu Xu, Jian Li, Xiaotian Zhang, Zhi Peng, Yajie Hu, Xinya Zhao, Kun Dong, Bei Zhang, Chan Gao, Xiaochen Zhao, Hui Chen, Jinping Cai, Yuezong Bai, Yu Sun, Lin Shen

**Affiliations:** 1 Key Laboratory of Carcinogenesis and Translational Research (Ministry of Education), Department of Gastrointestinal Oncology, Peking University Cancer Hospital & Institute, 52 Fucheng Road, Haidian District, Beijing, 100142 China; 2 Key Laboratory of Carcinogenesis and Translational Research (Ministry of Education), Department of Pathology, Peking University Cancer Hospital & Institute, 52 Fucheng Road, Haidian District, Beijing, 100142 China; 3 Medical Affairs, 3D Medicines, Inc, Shanghai, China

**Keywords:** dMMR/MSI-H gastric adenocarcinoma, PI3K-AKT-mTOR pathway, Tumor-infiltrating immune cell, Immune checkpoint inhibitor

## Abstract

**Background:**

A significant subset of mismatch repair-deficient (dMMR)/microsatellite instability-high (MSI-H) gastric adenocarcinomas (GAC) are resistant to immune checkpoint inhibitors (ICIs), yet the underlying mechanism remains largely unknown. We sought to investigate the genomic correlates of the density of tumor-infiltrating immune cells (DTICs) and primary resistance to ICI treatment.

**Methods:**

Four independent cohorts of MSI-H GAC were included: (i) the surgery cohort (*n* = 175) with genomic and DTIC data, (ii) the 3DMed cohort (*n* = 32) with genomic and PD-L1 data, (iii) the Cancer Genome Atlas (TCGA) cohort (*n* = 73) with genomic, transcriptomic, and survival data, and (iv) the ICI treatment cohort (*n* = 36) with pre-treatment genomic profile and ICI efficacy data.

**Results:**

In the dMMR/MSI-H GAC, the number of mutated genes in the PI3K-AKT-mTOR pathway (NMP) was positively correlated with tumor mutational burden (*P* < 0.001) and sensitivity to PI3K-AKT-mTOR inhibitors and negatively correlated with CD3^+^ (*P* < 0.001), CD4^+^ (*P* = 0.065), CD8^+^ (*P* = 0.004), and FOXP3^+^ cells (*P* = 0.033) in the central-tumor rather than invasive-margin area, and the transcription of immune-related genes. Compared to the NMP-low (NMP = 0/1) patients, the NMP-high (NMP ≥ 2) patients exhibited a poorer objective response rate (29.4% vs. 85.7%, *P* < 0.001), progression-free survival (HR = 3.40, *P* = 0.019), and overall survival (HR = 3.59, *P* = 0.048) upon ICI treatment.

**Conclusions:**

Higher NMP was identified as a potential predictor of lower DTICs and primary resistance to ICIs in the dMMR/MSI-H GAC. Our results highlight the possibility of using mutational data to estimate DTICs and administering the PI3K-AKT-mTOR inhibitor as an immunotherapeutic adjuvant in NMP-high subpopulation to overcome the resistance to ICIs.

**Supplementary Information:**

The online version contains supplementary material available at 10.1186/s12916-022-02327-y.

## Background

A mismatch repair-deficient/microsatellite instability-high (dMMR/MSI-H) phenotype often leads to a buildup of base-pair mismatches and slippage events over time [1–3], eventually causing high rates of insertions and deletions (indels) of nucleotides in microsatellites sequences [4]. The dMMR/MSI-H tumors are characterized by a high load of frameshift mutations and neoantigens, facilitating immune recognition and immune cell infiltration [5–7]. These infiltrating immune cells are commonly exhausted with upregulation of checkpoint proteins, including programmed death 1 (PD-1) and programmed death-ligand 1 (PD-L1), which serve as the targets of anti-PD-1/PD-L1 immunotherapy [8–10].

In theory, dMMR/MSI-H solid tumors should acquire favorable benefits from immune checkpoint inhibitor (ICI) treatment, yet nearly 40% of them had progressive disease as the best response [11, 12], suggesting the heterogeneity within this hypermutated subtype. Previous studies in MSI-H gastric adenocarcinoma (GAC) and colorectal cancer (CRC) suggested the association between mRNA-based clusters and survival of ICI therapy [13, 14]. However, little is known about the heterogeneity of immune-related features in MSI-H tumors, including the density of tumor-infiltrating immune cells (DTICs). A better understanding of the heterogeneity within these hypermutated tumor subsets and the unrecognized mechanism of resistance to ICIs is crucial for patient selection.

GAC is a major cause of cancer deaths worldwide [15]. It is one of the solid tumors with high microsatellite instability [11, 16–18], characterized by high abundant leukocyte infiltration, high proportion of activated immunophenotype, and strong correlation between clonal heterogeneity and immunophenotype [19]. Therefore, we were interested to see whether GAC patients with dMMR/MSI-H could be further dissected according to their immune-related features by depicting the mutational landscape of dMMR/MSI-H GAC and to investigate further the genomic correlates of immune cell infiltration and clinical benefits from ICI treatment.

## Methods

### Patients

In the surgery cohort (Additional file 1: Fig. S1) [20–29], 175 patients with resected primary gastric or gastro-esophageal junction (G/GEJ) cancer treated at Peking University Cancer Hospital and Institute from December 14, 2015, to November 13, 2019, were included. All tumors were evaluated by immunohistochemistry (IHC) to assess the expression of MMR proteins and were assigned a pathological tumor, node, and metastases (TNM) stage. This cohort was initially employed for a double-blinded technical validation of a 9-loci polymerase chain reaction (PCR) testing to identify MSI-H. Herein, we retrieved the patients' MSI status as determined by tissue-PCR of the Promega panel (BAT26, NR24, NR21, MONO27, and BAT25) and by next-generation sequencing (NGS) using a 733-gene panel incorporating 100 MSI loci (3D Medicines, Inc., Shanghai, China).

The 3DMed cohort consisted of 32 cases selected from the 51718 cases of the 3D Medicines database tested between January 6, 2017, and April 14, 2020. Cases were included only if they had PD-L1 expression data as evaluated by PD-L1 IHC 22C3 pharmDx (Dako, Inc.) and genomic profiling data by NGS using a 381-gene panel (Additional file 1: Fig. S1B).

The Cancer Genome Atlas (TCGA) cohort (*n* = 73) was obtained from the PanCancer stomach adenocarcinoma (STAD) dataset (Additional file 1: Fig. S1C), where patients were included if they had available mutational and transcriptional data, and an MSI-H phenotype diagnosed by the TCGA subtyping system [30]. Missense mutations in this cohort were evaluated by both Polyphen-2 and Sorting Intolerant From Tolerant to filter out potential benign alterations [31, 32]. Gene set enrichment analysis (GSEA) was performed to investigate the transcriptional difference.

The ICI treatment cohort (*n* = 36) was retrieved from the medical records of the Department of Gastrointestinal Oncology, Peking University Cancer Hospital and Institute, where patients with dMMR/MSI-H GAC received at least 1 cycle of any ICI treatment regardless of the agent's target (PD-1, PD-L1, or CTLA-4) from September 1, 2017, to January 31, 2020. These patients had their last follow-up before June 1, 2021, and NGS-based mutational data of pre-treatment tissue or plasma (Additional file 1: Fig. S1D). Moreover, ten patients had available data of pre-treatment peripheral blood lymphocyte subset counts via flow cytometry.

IHC, PCR, NGS, GSEA, drug sensitivity, flow cytometry, multiplex immunofluorescence, and assessment of tumor response were performed as previously published (Additional file 1: Supplementary Methods S1-7 and Additional file 1: Table S1-7) [20–29].

Human samples and clinical data were collected and used per the principles of the Declaration of Helsinki and approved by the Ethics committee of Peking University Cancer Hospital. All participants provided written informed consents. This report followed the Strengthening the Reporting of Observational Studies in Epidemiology (STROBE) reporting guideline.

### Statistical analysis

The significance with categorical variables was evaluated by Fisher's exact test. The significance with disease-free survival, progression-free survival (PFS), and overall survival (OS) was assessed by the Log-rank method. Univariable and multivariable Cox regression was implemented to calculate the hazard ratio (HR) of survival data. The significance with continuous variables was assessed by non-parametric tests (e.g., rank-sum tests, Spearman correlation test) or corrected parametric tests for variance correction (e.g., unpaired *t*-test with Welch’s correction). Receiver operating characteristic (ROC) curve and area under the curve (AUC) were used for seeking the best cutoff of the number of mutated genes in the PI3K-AKT-mTOR pathway (NMP) for predicting immunotherapeutic efficacy. All statistical analyses mentioned above were performed using IBM SPSS Statistics 22, and the graphs were drawn by GraphPad Prism 8 and RStudio (version 1.2.5042). We set the nominal significance level as 5%, and all 95% CIs were 2-sided.

## Results

### Clinicopathological and genomic features of dMMR/MSI-H GAC

**Fig. 1:**
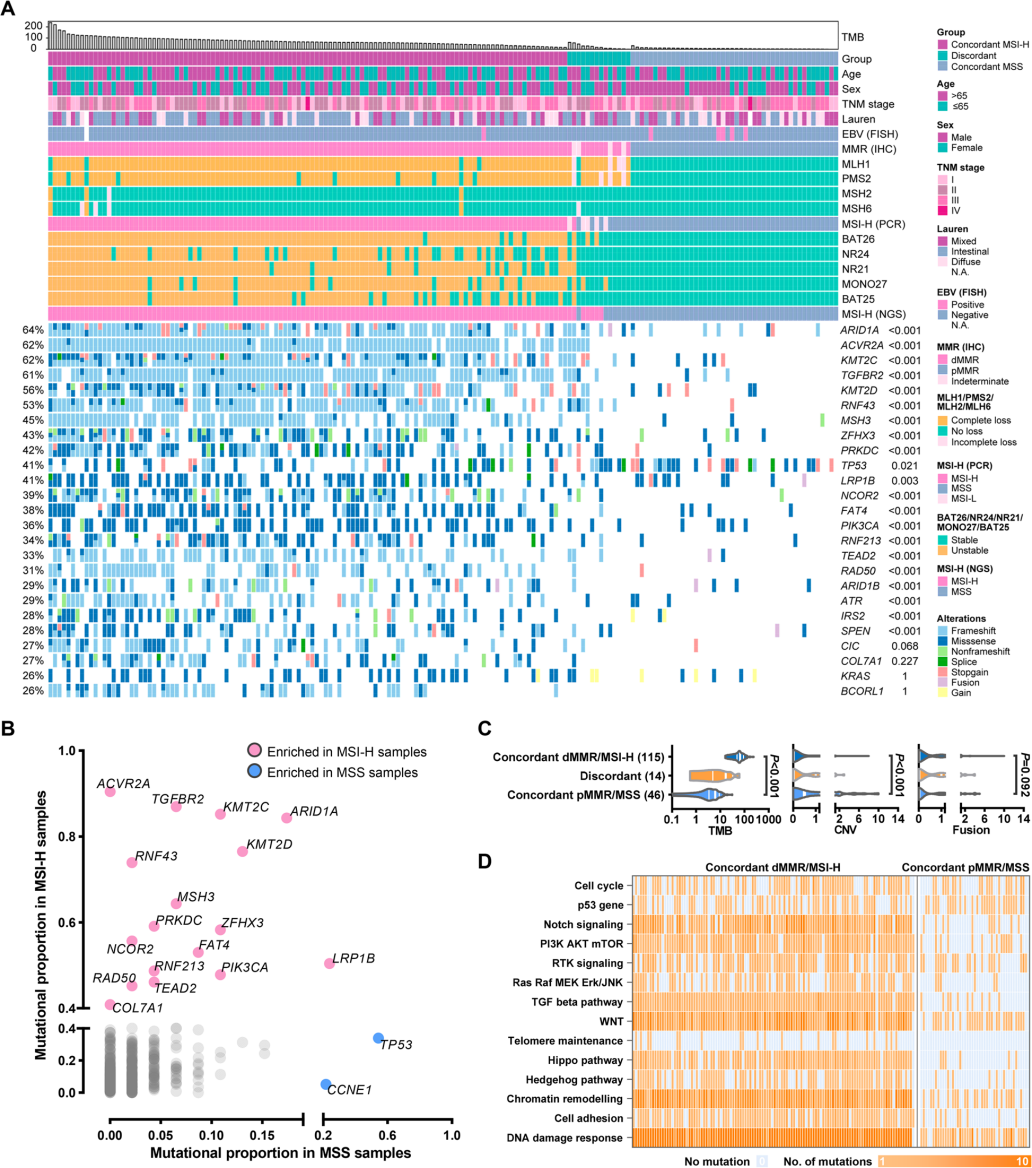
Clinicopathological and genomic characteristics of the surgery cohort. **A** Illustration of clinicopathological and genomic characteristics of the surgery cohort. Tumor mutational burden is shown in the upper panel. Basic clinicopathological characteristics are illustrated in the middle panel. Mutations with high frequencies are depicted in the lower panel. **B** Enriched genes mutated in concordant dMMR/MSI-H samples (highlighted in pink) or pMMR/MSS samples (highlighted in blue). **C** TMB, CNV, and fusion in concordant dMMR/MSI-H samples, discordant samples, and concordant pMMR/MSS samples. **D** Heatmap of the number of mutations in pre-specified pathways in concordant samples. Abbreviations: dMMR, mismatch repair-deficient; EBV, Epstein-Barr virus; FISH, fluorescence in situ hybridization; HER2, human epidermal growth factor receptor 2; IHC, immunohistochemistry; MLH1, MutL homolog 1; MMR, mismatch repair; MSH2, MutS Homolog 2; MSH6, MutS Homolog 6; MSI-H, microsatellite instability-high; MSI-L, microsatellite instability-low; MSS, microsatellite stability; N.A., not applicable; NGS, next-generation sequencing; PCR, polymerase chain reaction; PMS2, PMS1 Homolog 2; TMB, tumor mutational burden; CNV, copy number variation

The clinicopathological and genomic features of 175 resected samples from patients with primary G/GEJ adenocarcinoma are described in Additional file 1: Table S5 and Fig. 1A. IHC, PCR (5 loci), and NGS (100 loci) testing identified 115 concordant-dMMR/MSI-H samples, 46 concordant MMR-proficient (pMMR)/microsatellite stable (MSS) samples, and 14 discordant samples. The distribution of MSI score (by NGS testing), PCR score, and IHC result is displayed in Additional file 1: Fig. S2 and Table S6. In the 161 samples with concordant results of IHC and PCR testing, NGS was able to correctly detect MSI status with 100% sensitivity and 100% specificity. Furthermore, among the 14 samples where IHC result showed incomplete loss of MMR protein expression (e.g., loss of MSH6 expression in 50% of tumor cells and intact expression of MLH1/PMS2/MSH2) or was inconsistent with PCR results (e.g., IHC-dMMR but PCR-MSS), NGS identified 7 NGS-MSI-H cases with the highest variability of the tested microsatellites and the highest frameshift burden in the tested coding sequence (Additional file 1: Table S7). Taken together, NGS performed better in identifying MSI-H cases compared to IHC and PCR, especially when geographical heterogeneity of MMR protein expression is observed, or IHC and PCR results are inconsistent.

Among the concordant samples, dMMR/MSI-H status was associated with an early tumor stage (*P* = 0.050), intestinal type of Lauren classification (*P* = 0.058), lower HER2 expression (*P* = 0.034), and EBV negativity (*P* = 0.008, Additional file 1: Table S5). As for genetic aberration, mutations of *TP53* (*P* = 0.021) and *CCNE1* (*P* = 0.003) were enriched in pMMR/MSS samples, while the mutations in *ARID1A*, *ACVR2A*, *KMT2C*, *TGFBR2*, *KMT2D*, and *RNF43* genes were dominant in dMMR/MSI-H samples (*P* < 0.001) with mutational frequencies over 70% (Fig. 1B). Significantly higher tumor mutational burden (TMB) and the numbers of frameshift mutation, missense mutation, non-frameshift indel, stop-gain mutation, and splice site mutation were observed in concordant dMMR/MSI-H cases. On the contrary, copy number variation (CNV) and fusion were enriched in concordant pMMR/MSS cases (Fig. 1C and Additional file 1: Table S5).

Most dMMR/MSI-H samples carried mutations in the DNA damage response (99%), chromatin remodeling (99%), WNT (99%), TGFβ (98%), PI3K-AKT-mTOR (91%), Hippo (90%), and NOTCH (90%) pathways, while relatively lower mutational rates were discovered in the RTKs (73%), Hedgehog (68%), cell cycle (63%), Ras-Raf-MEK-ERK/JNK (62%), TP53 (56%), and telomere maintenance (19%) pathways (Fig. 1D). In the dMMR/MSI-H tumors with such high mutational burden and intratumoral heterogeneity, a singular mutation (e.g., *PTEN* mutation) can hardly reflect the functional alteration of the whole pathway (the PI3K-AKT-mTOR pathway) and the overall characteristics of the tumor. Therefore, we sought to define the number of mutated genes in the pre-specified pathway as a parameter representing the pathway’s potential changes and explore its correlation with the density of DTICs and response to ICI treatment in the dMMR/MSI-H GAC.

### Correlates of DTIC in dMMR/MSI-H GAC

Of the 115 concordant-dMMR/MSI-H cases, the evaluation of DTICs was missing in twelve cases (Additional file 1: Table S8), and another fourteen cases were excluded for prior neoadjuvant chemotherapy which could affect DTICs (Additional file 1: Table S9) [33–35]. Therefore, 89 samples were included for the following analysis of genomic correlates of DTICs.

**Fig. 2:**
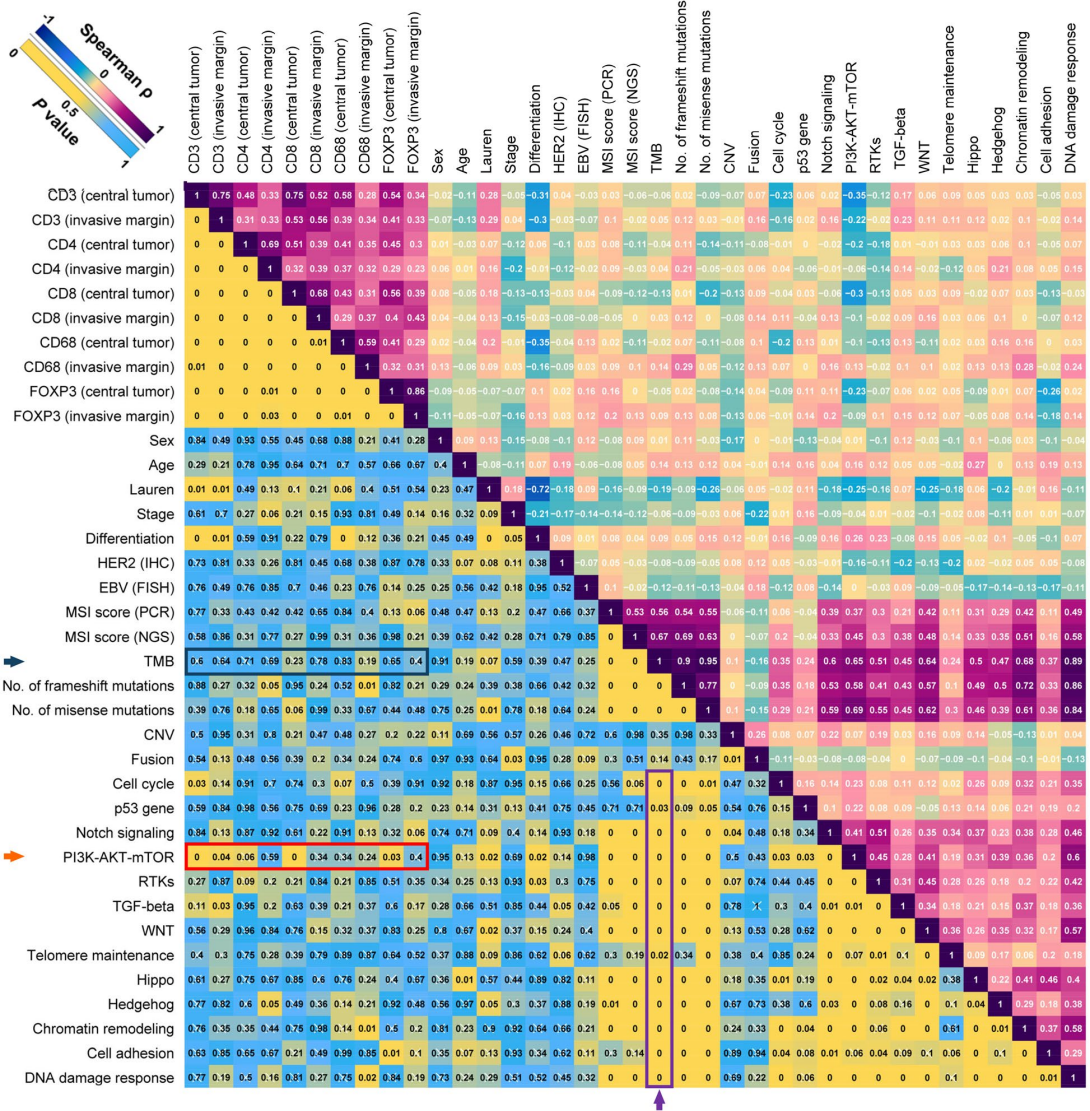
Correlates of immune cell infiltration in dMMR/MSI-H gastric adenocarcinomas without receiving neoadjuvant chemotherapy. Correlation matrix of NAC-naïve concordant dMMR/MSI-H samples in the surgery cohort. The values in the upper right part refer to the Spearman ρ, and the values in the lower left part indicate *P* values. The scales of colors are shown at the upper left corner. Abbreviations: CNV, copy number variation; dMMR, mismatch repair-deficient; EBV, Epstein-Barr virus; FISH, fluorescence in situ hybridization; HER2, human epidermal growth factor receptor 2; IHC, immunohistochemistry; MLH1, MutL homolog 1; MMR, mismatch repair; MSH2, MutS Homolog 2; MSH6, MutS Homolog 6; MSI, microsatellite instability; MSI-H, microsatellite instability-high; MSI-L, microsatellite instability-low; MSS, microsatellite stability; N.A., not applicable; NAC, neoadjuvant chemotherapy; NGS, next-generation sequencing; PCR, polymerase chain reaction; PMS2, PMS1 Homolog 2; RTK, receptor tyrosine kinase; TGF, transforming growth factor; TMB, tumor mutational burden

**Fig. 3:**
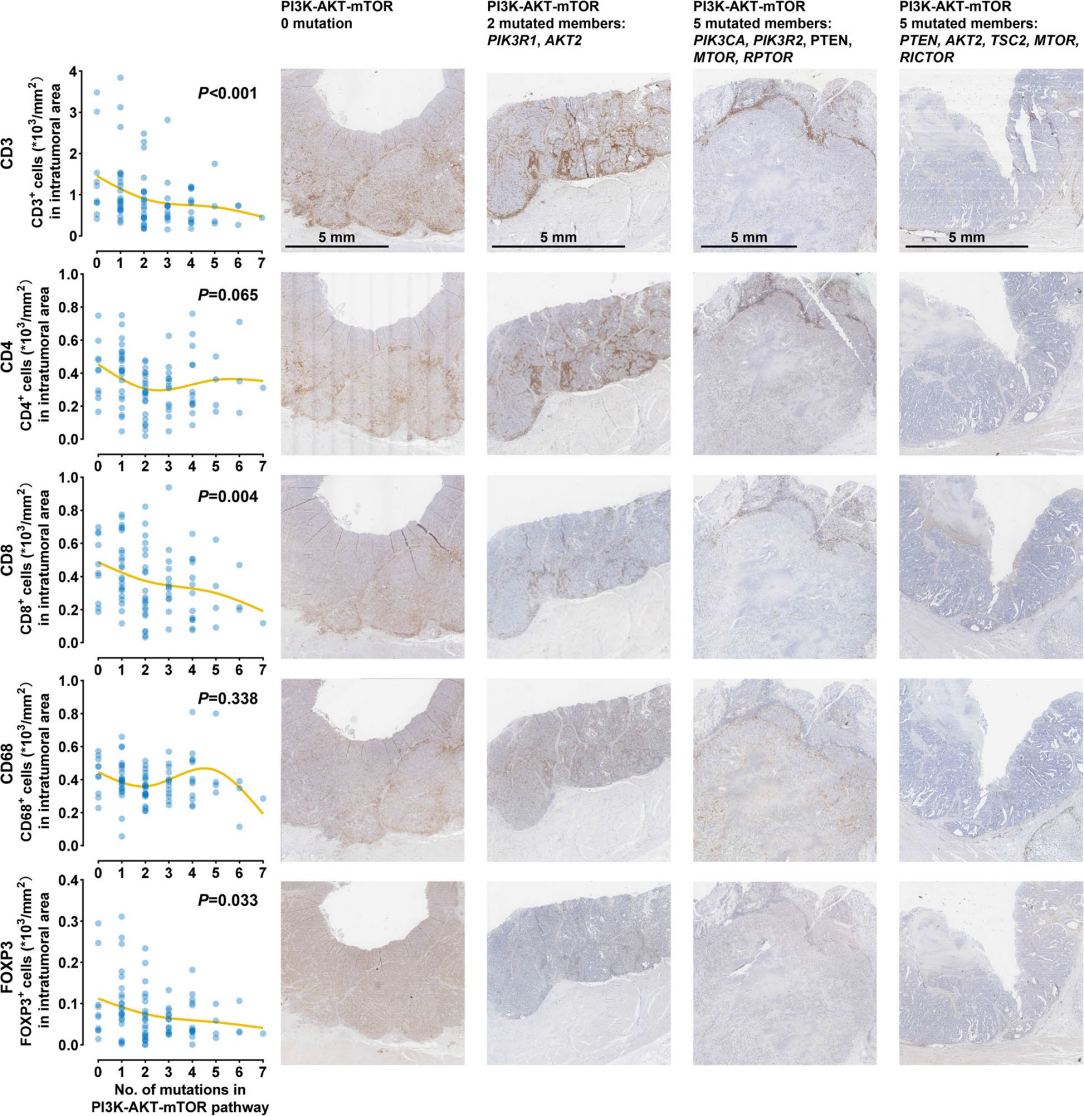
Association between NMP and immune cell infiltration in dMMR/MSI-H gastric adenocarcinomas without receiving neoadjuvant chemotherapy. Correlation of NMP with DTICs, including CD3^+^, CD4^+^, CD8^+^, and FOXP3^+^ cells in central tumor area (left part). Immunohistochemical staining of the representative samples for CD3, CD4, CD8, CD68, and FOXP3 (right part). Scale bar: 5 mm. Every blue point indicates the data of one sample, and the yellow lines are smoothing splines illustrating the trend of association between NMP and DTICs. Abbreviations: DTIC, density of tumor-infiltrating immune cell; dMMR, mismatch repair-deficient; MSI-H, microsatellite instability-high; NMP, number of mutated members of the PI3K-AKT-mTOR pathway

A comprehensive correlation matrix was created to seek the correlates of DTICs, including CD3^+^, CD4^+^, CD8^+^, CD68^+^, and FOXP3^+^ cells in central-tumor and invasive-margin areas (Fig. 2). The mutations of the members in the PI3K-AKT-mTOR pathway are illustrated in Additional file 1: Fig. S3. Among the clinicopathological and genomic characteristics, NMP exhibited the strongest negative correlation with DTICs, including CD3^+^ (*P* < 0.001), CD4^+^ (*P* = 0.065), CD8^+^ (*P* = 0.004), and FOXP3^+^ (*P* = 0.033) cells in the central tumor area (marked by a red arrow, Fig. 2). The correlations of NMP with central-tumor DTICs were markedly stronger than its correlations with invasive-margin DTICs, suggesting the potential difference of PI3K-AKT-mTOR function in central tumor and invasive margin. Sensitivity analysis further indicated the robustness of the results as mentioned above (Additional file 1: Table S10). The scatter diagrams of the above-mentioned results and representative images of immunohistochemical staining of tumor-infiltrating immune cells are displayed in Fig. 3.

Of note, TMB was strongly correlated with nearly all the numbers of mutated members in the pre-specified pathways (marked by a purple arrow, Fig. 2), including the PI3K-AKT-mTOR pathway, rather than DTICs (marked by a blue arrow, Fig. 2), consistent with previous results in MSI-H CRC [14, 36].

Given the correlations of NMP with DTICs, we next sought to investigate the associations of NMP with other potential predictors of immunotherapy, including TMB, PD-L1 expression, and immune-related mRNA signatures.

### Biological characteristics of the DTIC-enriched subtype with lower NMP

Higher TMB and PD-L1 levels were commonly associated with more clinical benefits from ICI treatment in MSS G/GEJ cancer [37–44]. Two recent retrospective studies with a small sample size suggested the association between TMB and immunotherapy efficacy in MSI-H GAC [45, 46]. Given these findings, we set out to evaluate TMB and PD-L1 expression in the DTIC-enriched subtype of MSI-H GAC with lower NMP.

**Fig. 4:**
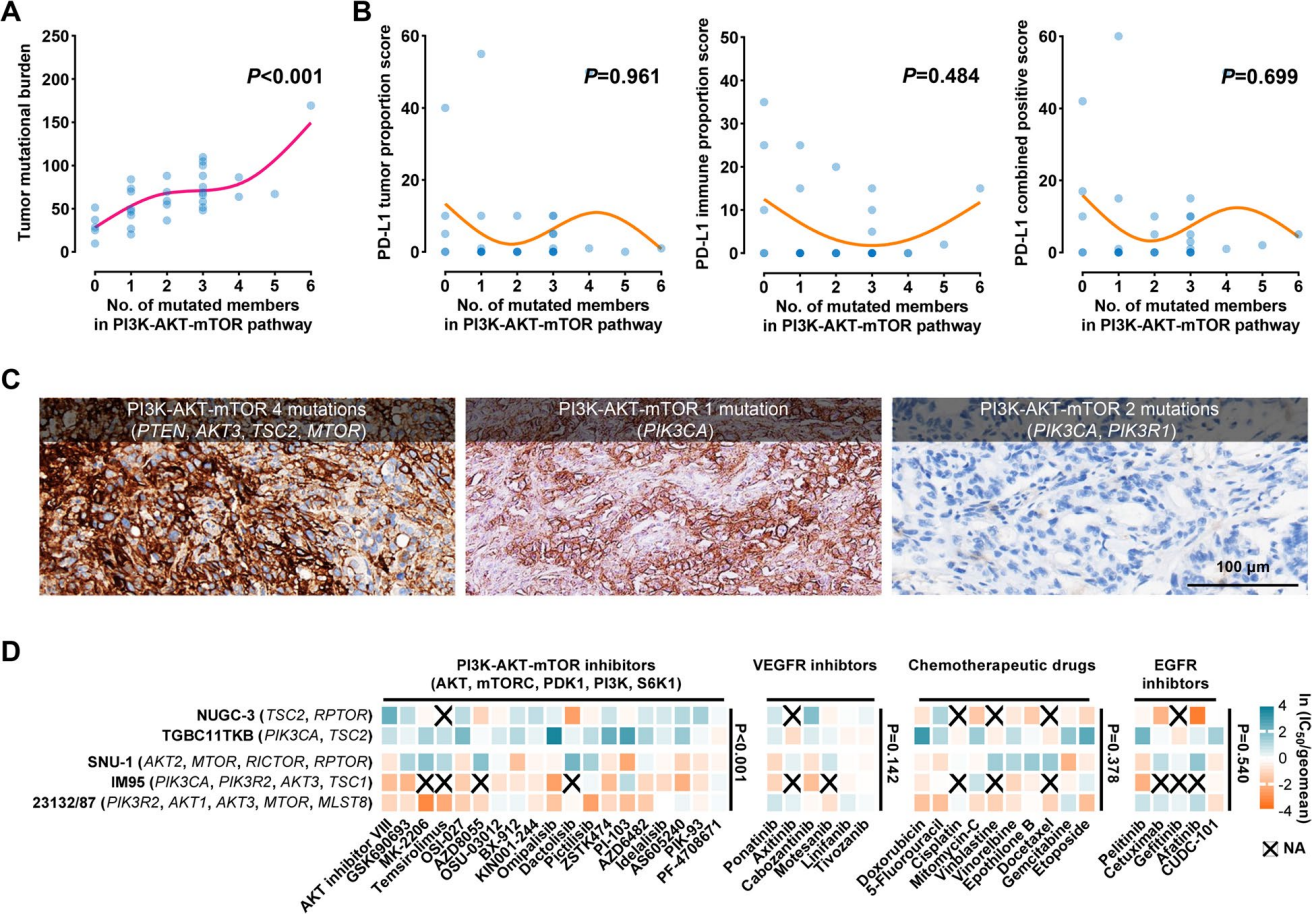
The associations of NMP with TMB, PD-L1, and drug sensitivity. **A** Correlation between NMP and TMB. Every blue point indicates the data of one sample, and the pink line is a smoothing spline illustrating the trend of association between NMP and TMB. **B** Correlation between NMP and PD-L1 expression (TPS, IPS, and CPS). Every blue point indicates the data of one sample, and the orange line is a smoothing spline illustrating the trend of association between NMP and PD-L1 expression. **C** Representative images of PD-L1 staining. **D** Drug sensitivity of the five MSI-H STAD cell lines. Abbreviations: CPS, combined positive score; IPS, immune proportion score; NMP, number of mutated members in the PI3K-AKT-mTOR pathway; PD-1, programmed death-1; PD-L1, programmed death-ligand 1; TMB, tumor mutational burden; TPS, tumor proportion score

We retrieved the data of 32 MSI-H GACs (primary lesion) with assessments of mutations and PD-L1 from the 3DMed database. Among these, lower NMP was correlated with lower TMB (*P* < 0.001, Fig. 4A), but not the PD-L1 expression in tumoral and immune cells (tumor proportion score [TPS]: *P* = 0.961; immune proportion score [IPS]: 0.484; combined positive score [CPS]: *P* = 0.699; Fig. 4B). Detailed characteristics and data are shown in Additional file 1: Table S11. The representative images of PD-L1 staining are shown in Fig. 4C, and the images of corresponding hematoxylin-eosin (HE) staining and positive/negative controls are enclosed in Additional file 1: Fig. S4.

Immune-related gene signatures were further explored in the TCGA cohort (characteristics are shown in Additional file 1: Table S12). Multiple significant enrichments of immune-related signatures were revealed in the PI3K-AKT-mTOR^WT^ group (Additional file 1: Fig. S5A), including MHC-II-mediated antigen presentation (*P* = 0.001), cross-presentation (*P* = 0.001), B cell receptor downstream signaling (*P* = 0.017), T cell receptor downstream signaling (*P* < 0.001), interferon signaling (*P* = 0.045), and PD-1 signaling (*P* = 0.006), while the activity of TGFβ signaling was decreased in the PI3K-AKT-mTOR^WT^ group (*P* < 0.001). Of note, despite the enrichment of PD-1 signaling in the PI3K-AKT-mTOR^WT^ group, the mRNA expression of *CD274* (PD-L1) was not higher in the PI3K-AKT-mTOR^WT^ group than in the PI3K-AKT-mTOR^mut^ group, suggesting that PD-L1 expression is irrelevant to PI3K-AKT-mTOR mutations (*P* = 0.341), consistent with the results in Fig. 4B. Similar enrichments in the PI3K-AKT-mTOR^WT^ group were observed in interleukin (IL) pathways, including IL-1 (*P* = 0.031), IL-12 (*P* = 0.014), and IL-12 family (*P* = 0.059, Additional file 1: Fig. S5B).

In addition to the immune-related gene signatures, we further assessed the signatures of NOTCH signaling. We previously reported that the NOTCH pathway’s downregulation was associated with a higher level of immune gene transcription and better immunotherapeutic efficacy in non-small cell lung cancer (NSCLC) [47]. Here, in the MSI-H GACs, the DTIC-enriched PI3K-AKT-mTOR^WT^ group displayed trends towards downregulation of NOTCH-related signatures, especially the ones concerning transcriptional impact (Additional file 1: Fig. S5C).

### PI3K-AKT-mTOR inhibitor efficacy of MSI-H GAC cell lines with different NMPs

Furthermore, to discover the association between PI3K-AKT-mTOR mutation and drug sensitivity, we retrieved the data of STAD cell lines from the cBioPortal (Cancer Cell Line Encyclopedia, Broad, 2019). Five of the 39 STAD cell lines were identified as MSI-H (NUGC-3, TGBC11TKB, SNU-1, IM95, and 23132/87). Among the five MSI-H GAC cell lines, NUGC-3 and TGBC11TKB had two mutations of the members of the PI3K-AKT-mTOR pathway, and SNU-1, IM95, and 23132/87 had no less than four member mutations. The mutations of the members in the PI3K-AKT-mTOR pathway and mutation sites in each cell line are shown in Additional file 1: Table S13. All mutations were pathogenic. The IC50 values of every cell line for the PI3K-AKT-mTOR inhibitors (targeting AKT/mTOR/PDK1/PI3K/S6K1), the inhibitors targeting VEGFR or EGFR, and chemotherapeutic drugs were presented in Additional file 1: Table S14. The PI3K-AKT-mTOR inhibitors exhibited lower IC_50_ in the SNU-1, IM95, and 23132/87 cell lines (*P* < 0.001) than in NUGC-3 and TGBC11TKB cell lines, and the inhibitors targeting VEGFR (*P* = 0.142) or EGFR (*P* = 0.540) and chemotherapeutic drugs (*P* = 0.378) showed no significant difference of sensitivity (Fig. 4D). Taken together, these results indicate that the MSI-H STADs with high NMP might benefit more from PI3K/AKT/mTOR inhibitors compared to the ones with low NMP.

### Immunotherapy efficacy of the DTIC-enriched subtypes with lower NMP

The subtype with lower NMP was characterized by higher DTICs and immune-related gene transcription (potentially associated with better ICI response) [42, 48] and lower TMB (potentially associated with poorer ICI response) in MSI-H GAC [37, 38, 49]. Given these opposite predictive values, we sought to investigate the ICI efficacy of this subtype.

**Fig. 5:**
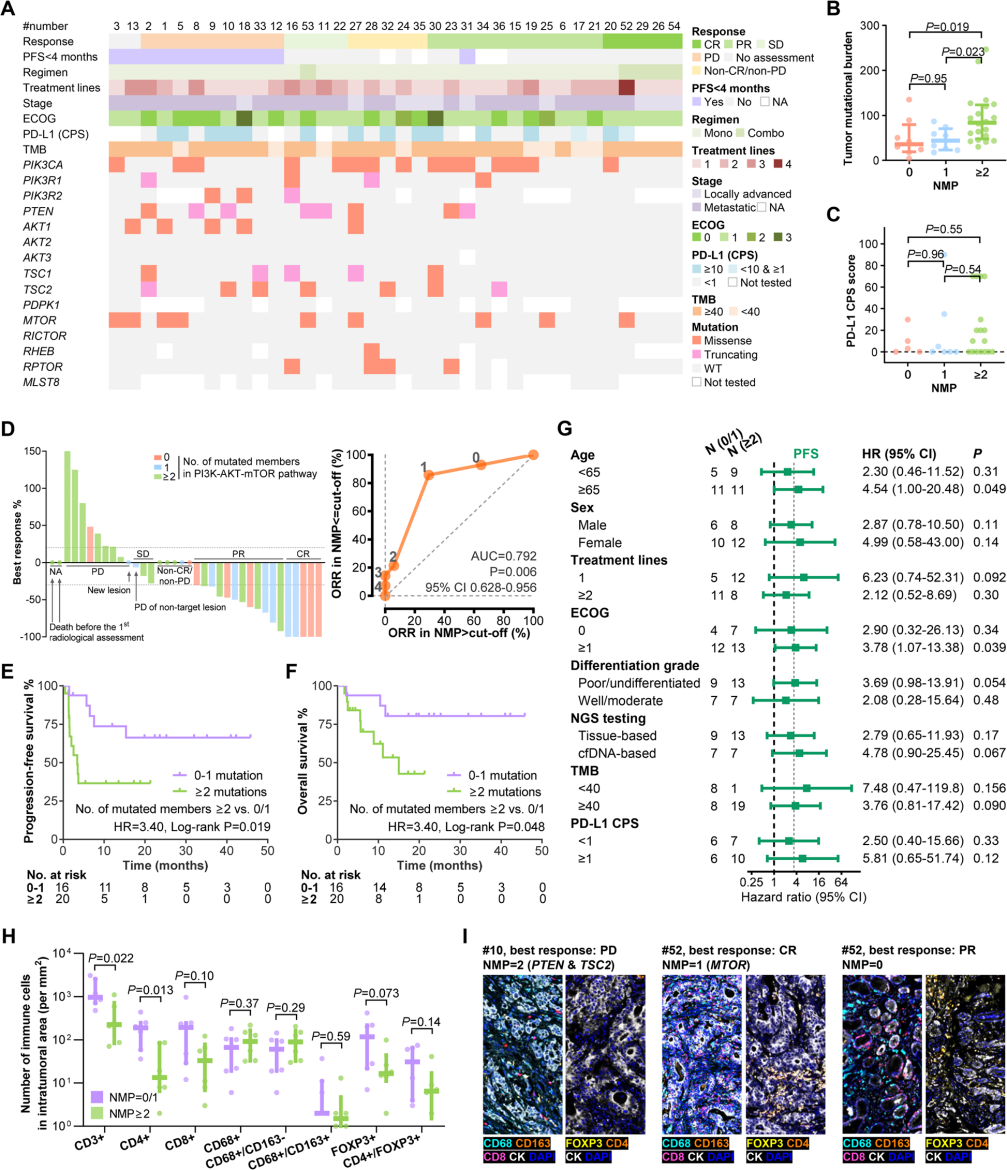
Association between NMP and response to ICI in dMMR/MSI-H G/GEJ adenocarcinomas. **A** Basic clinicopathological characteristics and mutations of the PI3K-AKT-mTOR pathway of the ICI treatment cohort. **B**, **C** Association of NMP with tumor mutational burden (**B**) and PD-L1 CPS score (**C**). **D** Bar plot illustrating best response and ROC curve of the NMP to predict ORR. The NMP of each patient is labeled with red (NMP, 0), blue (NMP, 1), and green (NMP > 1). **E**, **F** Kaplan-Meier curves of PFS (**E**) and OS (**F**) in the NMP-low (0/1) and NMP-high (> 1) patients. **G** Subgroup analysis of PFS. **H** Association between NMP and DTICs. **I**. Representative images of DTICs. Abbreviations: AUC, area under curve; CI, confidence interval; CPS, combined positive score; CR, complete response; DTIC, density of tumor-infiltrating immune cells; ECOG, Eastern Cooperative Oncology Group; HR, hazard ratio; NA, not applicable; ORR, objective response rate; OS, overall survival; PD, progressive disease; PD-L1, programmed death-ligand 1; PFS, progression-free survival; PR, partial response; SD, stable disease; WT, wildtype

In total, 36 patients with locally advanced or metastatic concordant-dMMR/MSI-H G/GEJ adenocarcinoma were included. The key baseline characteristics, individual response to ICI treatment, and mutational events of the members of the PI3K-AKT-mTOR pathway are illustrated in Fig. 5A and Additional file 1: Table S15. The best responses are shown in Fig. 5D. To explore the optimal cutoff of the NMP for predicting immunotherapy efficacy in dMMR/MSI-H G/GEJ adenocarcinoma, a ROC curve was plotted based on the objective response in patients with evaluable target lesion (*n* = 31). The AUC of NMP was significantly higher than 0.5 (AUC = 0.792, 95% CI 0.628-0.956, *P* = 0.006, Fig. 5D), suggesting the feasibility of using NMP to predict response to ICIs. The optimal cutoff was set as 1 when the largest Youden index was achieved. In the NMP-high patients (NMP ≥ 2), objective response rate (ORR) was 29.4%, and 4-month PFS rate was 35.3%, while in the NMP-low patients (NMP = 0/1), ORR and 4-month PFS rate were significantly higher as 85.7% (*P* = 0.002) and 93.3% (*P* = 0.001), respectively (Table 1).

**Table 1:** Response to immunotherapy in the ICI treatment cohort

	Total (*n* = 36)	NMP = 0/1 (*n* = 16)	NMP ≥ 2 (*n* = 20)	*P* value
CR	5 (13.9%)	5 (31.3%)	0 (0.0%)	
PR	12 (33.3%)	7 (43.8%)	5 (25.0%)	
Non-CR/non-PD	5 (13.9%)	2 (12.5%)	3 (15.0%)	
Stable disease	3 (8.3%)	1 (6.3%)	2 (10.0%)	
PD	9 (25.0%)	1 (6.3%)	8 (40.0%)	
No assessment^a^	2 (5.6%)	0 (0%)	2 (10.0%)	
ORR in modified population (*n* = 31)^b^	54.8%	85.7%	29.4%	0.002
4-month PFS rate (*n* = 32)^c^	62.5%	93.3%	35.3%	0.001

*Abbreviations*: *CR* complete response, *ORR* objective response rate, *PD* progressive disease, *PFS* progressive-free disease, *PR* partial response

^a^No assessment represents the patients who had a baseline assessment but no post-baseline assessment at the time of the data cutoff date, due to death before the first post-baseline radiologic imaging assessment

^b^Modified population represents the patients with evaluable target lesion

^c^Four patients who have been followed up for less than 4 months and have not yet progressed were excluded from the analysis of 4-month PFS rate

Consistent with our previous findings, the NMP-high group exhibited higher TMB than the NMP-low group (Fig. 5B), and NMP was not associated with PD-L1 CPS (Fig. 5C). In addition, the ROC curves based on the objective response demonstrate that TMB (AUC = 0.582, 95% CI 0.377–0.787, *P* = 0.44) and PD-L1 CPS (AUC = 0.529, 95% CI 0.291–0.767, *P* = 0.82) were not associated with the response to ICI treatment in the dMMR/MSI-H G/GEJ adenocarcinomas (Additional file 1: Fig. S6).

The clinical characteristics were comparable between the NMP-high and NMP-low groups (Additional file 1: Table S15). The median time to progression or death was 3.4 months in the NMP-high patients versus not reached in the NMP-low patients (HR = 3.40, 95% CI 1.16–10.00, Log-rank *P* = 0.019, Fig. 5E). Similarly, shorter median OS was observed in the NMP-high patients as 15.0 months, compared with not reached in the NMP-low patients (HR = 3.59, 95% CI 0.94-13.78, Log-rank *P* = 0.048, Fig. 5F). The maturity of OS was 30.6%, contributing to the slight difference between the results of Cox regression and Log-rank statistics (*P* = 0.063 and 0.048, respectively), and therefore, we set PFS as the major outcome in the following analyses.

Despite that higher TMB was observed in the NMP-high group, we found that the predictive effect of NMP on PFS was similar in the TMB-high (≥ 40 mutations/Mb) and TMB-low (< 40 mutations/Mb) subgroups (Fig. 5G). This cutoff of TMB (40) was selected based on the first study pointing out the predictive effect of TMB in MSI-H gastrointestinal tumors [49]. Further subgroup analysis suggests the consistent predictive effects of NMP in patients with different characteristics, including treatment lines, ECOG, differentiation grade, PD-L1 CPS, and NGS testing technique (Fig. 5G). Univariable analyses of PFS and OS did not reveal potential predictive biomarker other than NMP, and the multivariable analyses identified NMP as a predictor independent of TMB and PD-L1 for PFS (multivariable HR = 5.99, 95% CI 1.21–29.61, *P* = 0.028) and OS (multivariable HR = 11.88, 95% CI 1.30–108.4, *P* = 0.028) (Additional file 1: Table S16). Consistent with the results in Fig. 3, compared to the NMP-high subgroup, higher DTICs, including CD3^+^ (*P* = 0.022) CD4^+^ (*P* = 0.013), CD8^+^ (*P* = 0.10), and FOXP3^+^ cells (*P* = 0.073) were observed in the NMP-low subgroup by multiplex immunofluorescence, while CD68^+^ macrophage (*P* = 0.37), M1 macrophage (CD68^+^/CD163^−^, *P* = 0.29), and M2 macrophage (CD68^+^/CD163^+^, *P* = 0.59) were not significantly differed (Fig. 5H, I).

A previous retrospective study involving 45 dMMR/MSI-H gastrointestinal tumors (18 gastric tumors) suggests that the mutation of *PTEN*, a key member of the PI3K-AKT-mTOR pathway, was associated with poor response to ICIs [45]. Given this, we first tried to validate this result in our 36 cases and only observed non-significant trends in PFS (*P* = 0.34, Additional file 1: Fig. S7A) and OS (*P* = 0.29, Additional file 1: Fig. S7B). Moreover, in the 28 *PTEN*^WT^ cases, NMP-high remained as an indicator of poorer PFS (HR = 5.98, 95% CI 1.47–24.22, *P* = 0.005, Additional file 1: Fig. S7C) and OS (HR = 5.95, 95% CI 1.09–32.47, *P* = 0.022, Additional file 1: Fig. S7D). Taken together, NMP, as a predictor concerning multiple key members of the PI3K-AKT-mTOR pathway, performed more robustly and powerfully than a singular *PTEN* mutation.

In addition, to exclude the possibility that the association between NMP and ICI efficacy was impacted by the pre-treatment immune cell concentration in peripheral blood (reflecting systemic immunity), we detected the correlation between NMP and the pre-treatment status of peripheral blood lymphocyte subsets and observed no significant correlations (Additional file 1: Fig. S8). Moreover, PI3K-AKT-mTOR^mut^ was not associated with the disease-free survival (*P* = 0.37) and OS (*P* = 0.37) in the MSI-H GAC cases of the TCGA database (Additional file 1: Fig. S9). Collectively, NMP-high was identified as a predictive rather than prognostic biomarker, associated with inferior clinical benefit from ICI treatment in patients with dMMR/MSI-H G/GEJ adenocarcinoma.

## Discussion

This study represents one of the first reports to dissect further dMMR/MSI-H GAC from the aspects including genome, transcriptome, DTIC, and response to ICI treatment. NMP-low was correlated with lower TMB, higher DTICs, greater transcription of immune-related genes, and superior outcome from ICI treatment.

The status of dMMR/MSI-H could be determined by IHC, PCR, and NGS, wherein IHC is the first test choice. In case of doubt of IHC, a confirmatory molecular analysis must be performed. For molecular analysis of dMMR/MSI-H, the NCCN Guidelines for gastric cancer indicate that MSI can be assessed by PCR to measure gene expression levels of microsatellite markers (i.e., BAT25, BAT26, MONO27, NR21, NR24) [50]. The panel with these five poly-A repeats has been widely used in important clinical trials, including Keynote-016 and Keynote-059 [42, 51]. Based on these findings, we adopted the panel with five poly-A mononucleotide repeats for determining MSI status. For the surgery cohort which was used to explore associations between specific pathways and DTICs, only samples with IHC-dMMR, PCR-MSI-H and NGS-MSI-H were included. The strict criteria made the following analysis reliable and convincing. In addition, among the three methods, NGS demonstrates the perfect accuracy and the potential superiority in identifying MSI-H cases, especially when IHC and PCR results are inconsistent.

DTIC is a crucial predictive biomarker of ICI efficacy [52]. Higher TMB was generally correlated with greater DTICs, due to the immune activation via MHC-mediated neoantigen presentation [53]. However, within the hypermutated MSI-H subtype with numerous neoantigens, the impact of more mutations on greater immune activation might have reached a plateau. A similar phenomenon has been reported in MSI-H CRC [14, 36]. Consistently, we found no correlation between TMB and DTICs in dMMR/MSI-H GAC. As for the association between TMB and ICI benefit in MSI-H gastrointestinal cancers, Chida et al. set the cutoff as 10 mutations/Mb to identify TMB-low cases who exhibited a poor response to ICI treatment in their MSI-H gastrointestinal cohort [45]. TCGA network analyzed the pan-gastrointestinal adenocarcinomas, including MSS and MSI-H tumors, and defined the MSI subtype by TMB > 10 mutations/Mb, indel burden > 1, and indel/SNV ratio > 1/150 [54]. Based on this, the extremely low TMB (< 10 mutations/Mb) in some MSI-H cases in the study by Chida et al. is more likely to indicate the false positivity of their assessments of MSI-H by PCR and/or dMMR by IHC. In addition, Kwon et al. found that TMB < 26mutations/Mb was associated with poorer PFS on pembrolizumab in a small cohort of MSI-H GAC (*n* = 15) [46]. However, in our cohort involving 36 G/GEJ adenocarcinomas patients with concordant MSI-H status by NGS and IHC assessments, the TMB-based AUC for ICI response was merely 0.582 (*P* = 0.44), and no cutoff value can dissect the patients into subgroups with distinct survival outcomes. These findings suggest that TMB might not be a valid predictive factor for the benefit of ICI treatment in MSI-H GAC. Instead, some specific mutated genes and their functional impacts on the DTICs and expression of immune-related genes might be more important in determining the immunotherapy efficacy.

In the tumors with fewer mutations (e.g., MSS and *POLD1*/*POLE*-wildtype GAC), one singular genetic aberration may induce crucial influence. However, in the hypermutated tumors (e.g., MSI-H or *POLD1*/*POLE*-mutant tumors), the effect of a single mutation might be diluted. Therefore, in this study, we investigated the association between tumor immune microenvironments and specific pathways instead of specific gene alterations.

The PI3K-AKT-mTOR pathway controls most hallmarks of cancer, including cell cycle, motility, survival, metabolism, and genomic instability. Mutations of the members in the PI3K-AKT-mTOR pathway generally activate this signaling [55]. The activation-induced extensive carcinogenicity of this pathway has prompted the development of therapeutic reagents to inhibit this pathway. Of note, this signaling has also been well-documented to be involved in the tumor immune microenvironments, regulating the secretion of immunosuppressive cytokines, the expression of PD-L1, the infiltration of myeloid-derived suppressor cells, regulatory T cells and CD8^+^ T cell into tumor tissues, and the development of memory T cells [56]. In this study, it was also found that multiple immune-related signatures were significantly enriched and the activity of TGFβ signaling was decreased in the PI3K-AKT-mTOR^WT^ group compared to those in the PI3K-AKT-mTOR^mut^ group. Some research showed that pharmacological inhibition of this pathway not only restored immune-related signal transduction and improved antigen presentation but also increased DTICs, facilitating immune recognition on tumor cells [57–59]. The degree of pathway’s activation was further assessed by the number of mutated genes. The results from the surgery cohort showed that higher NMP was associated with poorer immune cell infiltration. Analysis of drug sensitivity in MSI-H GAC cell lines revealed higher sensitivities to PI3K-AKT-mTOR inhibitors in the cell lines with higher NMP. These findings were further validated in MSI-H GAC patients treated with ICIs, wherein NMP-low patients (NMP = 0/1) were DTIC-enriched and had better response and longer survival durations on ICI treatment compared to those with high NMP (NMP ≥ 2). Taken together, higher NMP might be one of the mechanisms underlying immune evasion and primary resistance to immunotherapy in dMMR/MSI-H gastric adenocarcinomas. In addition, the impacts of the PI3K-AKT-mTOR pathway on PD-1 signaling might be independent of regulating PD-L1 expression, based on our negative findings and a previous report in melanoma [60].

Preclinical data suggest the upregulation of DTIC via administrating pan-PI3K inhibitor (BKM120), and its synergistic effect with anti-PD-1 in the mouse model bearing breast cancer or muscle-invasive bladder cancer patient-derived xenograft [58, 59]. Currently, multiple trials are ongoing to evaluate the anti-tumor activity of this combination. For example, the NCT03673787 trial (ipatasertib plus atezolizumab) enrolls patients with pathogenic mutations in *PIK3CA*, *AKT1*, and *AKT2* identified by NGS, or PTEN loss by IHC. Several treatment schedules for the combination of ipatasertib and atezolizumab have been proposed in patients with advanced solid tumors [61–63]. These treatment schedules mainly fall into two categories: (i) ipatasertib and atezolizumab are co-administrated directly, and (ii) ipatasertib is given priority for a period of time, followed by combining with atezolizumab. Given our findings and previous studies showing that NMP was negatively correlated with DTICs, and the treatment with 2 weeks of single-agent ipatasertib induced the decrease of CD4^+^FOXP3^+^ regulatory T cells in the tumor microenvironment [61], there may be more advantages to the second category of treatment schedules. Furthermore, a manageable safety profile is observed with ipatasertib plus atezolizumab across multiple tumor types [61–63]. Taken together, despite the inferior benefit from ICIs in the NMP-high cases, combination therapy with PI3K-AKT-mTOR inhibitors might be a promising choice to increase DTICs and enhance immunotherapy efficacy in this population.

Although DTICs were associated with ICI efficacy [52], it has not been widely used in clinical practice due to the lack of standard evaluation method and sufficient tumor samples after the multiple recommended tissue-based assessments, including protein expression of HER2, MMR, and PD-L1 by IHC, and Epstein-Barr virus-encoded small RNA by in situ hybridization in gastric cancer. Under these circumstances, NGS testing of circulating tumor DNA (ctDNA), as a technique providing mutational data with high credibility and validity, may be an alternative method to evaluate the DTICs in tumor tissues by leveraging the correlations between DTICs and genetic aberrations. Meanwhile, given the high level of intratumoral heterogeneity in GACs, ctDNA-based NGS testing could provide more reliable MSI status, TMB, and genetic indicators for precision therapy (e.g., the fusion of *NTRK*) than tissue-based testing.

As for limitations, first, the DTIC results are based on several common tumor-infiltrating immune cell subsets used for evaluation of tumor microenvironment, and these results detected by IHC might not be as ample as the one calculated in silico from RNA-seq data. However, IHC provides histological illustration, enabling pathologists to score the DTIC in different regions. In the present study, we separately assess the DTICs in the central tumor area and invasive margin area. The correlations of NMP with invasive-margin DTICs were much weaker than its correlations with central-tumor DTICs, suggesting the potential distinction of the impacts of PI3K-AKT-mTOR on DTICs in the tumor center and margin. Additionally, given the complexity and trickiness of the tumor microenvironments, the correlations of NMP with the tumor-infiltrating lymphocytes need to be further explored in more tumor-infiltrating immune cell subsets. Second, the retrospective setting of our study may introduce biases, but this limitation has been minimized by the balanced characteristics in the NMP-high and NMP-low subgroups, and the implementation of subgroup analysis and multivariable analysis to exclude the confounding impacts from these variables. NMP held great promise by its broad applicability for the high predictive value of ICI efficacy regardless of treatment lines, ECOG, pathology, NGS testing technique, TMB, and PD-L1 CPS, making it meaningful for patient selection.

## Conclusions

Our findings demonstrate the heterogeneity of genotypes, DTICs, immune-related signatures, and immunotherapy efficacy in the dMMR/MSI-H GACs. Higher NMP, identified as a genomic correlate of lower DTICs in this population, might serve as a potential predictor of intrinsic resistance to anti-PD-(L)1 treatment. Additional studies are warranted to determine the synergistic effect of PI3K-AKT-mTOR inhibitors to overcome the resistance to ICI treatment in the NMP-high dMMR/MSI-H G/GEJ adenocarcinomas.

## Supplementary Information

**Additional file 1. Fig. S1.** Work flow and patient selection. **Fig. S2.** Association between the results of IHC, PCR, and NGS testing for identifying dMMR/MSI-H gastric adenocarcinoma. **Fig. S3.** Mutations of PI3K-AKT-mTOR pathway of the concordant MSI-H cases in the surgery cohort. **Fig. S4.** Representative images of hematoxylin-eosin and PD-L1 staining of MSI-H STAD samples in the 3DMed cohort. **Fig. S5.** GSEA of gene signatures related to immune activation, interleukin pathways, and NOTCH signaling in comparisons between samples with or without mutation in PI3K-AKT-mTOR pathway. **Fig. S6.** ROC curves illustrating the association of response rate with TMB and PD-L1 CPS. **Fig. S7.** Predictive effect of PTEN mutation and NMP in *PTEN*^*WT*^ dMMR/MSI-H gastric adenocarcinomas. **Fig. S8.** Association between mutations in PI3K-AKT-mTOR pathway and concentration of peripheral blood immune cells in patients with dMMR/MSI-H G/GEJ adenocarcinoma in the ICI treatment cohort. **Fig. S9.** Prognostic effect of the genetic aberration in PI3K-AKT-mTOR pathway in the TCGA cohort. **Table S1.** List of the genes in the 3DMed 733-gene panel. **Table S2.** Members of the analyzed signaling pathways in the surgery cohort. **Table S3.** Antibodies used in flow cytometry. **Table S4.** List of gene signatures in GSEA. **Table S5.** Clinicopathological and genomic characteristics in the surgery cohort. **Table S6.** Association between the results of IHC, PCR, and NGS testing for identifying dMMR/MSI-H gastric adenocarcinoma. **Table S7.** Genomic characteristics of the discordant samples. **Table S8.** Clinicopathological characteristics of concordant dMMR/MSI-H samples according to the evaluation of immune infiltration in the surgery cohort. **Table S9.** Clinicopathological characteristics of dMMR/MSI-H samples with evaluation of immune infiltration according to the history of neoadjuvant chemotherapy in the surgery cohort. **Table S10.** Sensitivity analysis of the correlation between mutations in PI3K-AKT-mTOR pathway and immune cell infiltration. **Table S11.** Detailed information of the MSI-H gastric adenocarcinoma samples evaluated by both 381-gene panel and PD-L1 kit retrieved from the 3DMed database. **Table S12.** Clinicopathological characteristics of MSI-H STAD according to the genetic aberration in PI3K-AKT-mTOR pathway in the TCGA cohort. **Table S13.** The mutations of the members in the PI3K-AKT-mTOR pathway and mutation sites in the five MSI-H GAC cell lines. **Table S14.** The IC50 values of every cell line for the PI3K-AKT-mTOR inhibitors, the inhibitors targeting VEGFR or EGFR, and chemotherapeutic drugs. **Table S15.** Baseline characteristics of the patients with dMMR/MSI-H G/GEJ adenocarcinoma in the ICI treatment cohort. **Table S16.** Univariable and multivariable analysis of PFS and OS in the ICI treatment cohort.

## Data Availability

The datasets supporting the conclusions of this article are included within the article and its additional files.

## Abbreviations

dMMR: Mismatch repair-deficient
MSI-H: Microsatellite instability-high
GAC: Gastric adenocarcinomas
DTICs: The density of tumor-infiltrating immune cells
ICI: Immune checkpoint inhibitor
TCGA: The Cancer Genome Atlas cohort
NMP: The number of mutated genes in the PI3K-AKT-mTOR pathway
PD-L1: Programmed death-ligand 1
PD-1: Programmed death 1
CRC: Colorectal cancer
G/GEJ: Gastric or gastro-esophageal junction
TNM: Tumor, node, and metastases
PCR: Polymerase chain reaction
NGS: Next-generation sequencing
HR: Hazard ratio
PFS: Progression-free survival
OS: Overall survival
ROC: Receiver operating characteristic
CNV: Copy number variation
