# Supplementary material for: Mutations of PI3K-AKT-mTOR pathway as predictors for immune cell infiltration and immunotherapy efficacy in dMMR/MSI-H gastric adenocarcinoma
Source: BMC Med. 2022 Apr 21;20:133. doi: 10.1186/s12916-022-02327-y (PMC9022268; doi:10.1186/s12916-022-02327-y)
Supplement: Supplementary file 1 — Additional file 1. Fig. S1. Work flow and patient selection. Fig. S2. Association between the results of IHC, PCR, and NGS testing for identifying dMMR/MSI-H gastric adenocarcinoma. Fig. S3. Mutations of PI3K-AKT-mTOR pathway of the concordant MSI-H cases in the surgery cohort. Fig. S4. Representative images of hematoxylin-eosin and PD-L1 staining of MSI-H STAD samples in the 3DMed cohort. Fig. S5. GSEA of gene signatures related to immune activation, interleukin pathways, and NOTCH signaling in comparisons between samples with or without mutation in PI3K-AKT-mTOR pathway. Fig. S6. ROC curves illustrating the association of response rate with TMB and PD-L1 CPS. Fig. S7. Predictive effect of PTEN mutation and NMP in PTENWT dMMR/MSI-H gastric adenocarcinomas. Fig. S8. Association between mutations in PI3K-AKT-mTOR pathway and concentration of peripheral blood immune cells in patients with dMMR/MSI-H G/GEJ adenocarcinoma in the ICI treatment cohort. Fig. S9. Prognostic effect of the genetic aberration in PI3K-AKT-mTOR pathway in the TCGA cohort. Table S1. List of the genes in the 3DMed 733-gene panel. Table S2. Members of the analyzed signaling pathways in the surgery cohort. Table S3. Antibodies used in flow cytometry. Table S4. List of gene signatures in GSEA. Table S5. Clinicopathological and genomic characteristics in the surgery cohort. Table S6. Association between the results of IHC, PCR, and NGS testing for identifying dMMR/MSI-H gastric adenocarcinoma. Table S7. Genomic characteristics of the discordant samples. Table S8. Clinicopathological characteristics of concordant dMMR/MSI-H samples according to the evaluation of immune infiltration in the surgery cohort. Table S9. Clinicopathological characteristics of dMMR/MSI-H samples with evaluation of immune infiltration according to the history of neoadjuvant chemotherapy in the surgery cohort. Table S10. Sensitivity analysis of the correlation between mutations in PI3K-AKT-mTOR pathway and immune cell infiltrati [file 12916_2022_2327_MOESM1_ESM.docx]

**Online-Only Supplement**

Supplement to: X. Wang, Z. Wang, Y. Xu, et al. Mutations of PI3K-AKT-mTOR pathway as predictors for immune cell infiltration and immunotherapy efficacy in dMMR/MSI-H gastric adenocarcinoma.

**TABLE OF CONTENTS**

[Supplementary Method S1. Immunohistochemistry 3](#_Toc97212033)

[Supplementary Method S2. PCR testing of microsatellites 3](#_Toc97212034)

[Supplementary Method S3. NGS testing 4](#_Toc97212035)

[Supplementary Method S4. Gene set enrichment analysis (GSEA) 4](#_Toc97212036)

[Supplementary Method S5. Analysis of drug sensitivity of STAD cell lines 5](#_Toc97212037)

[Supplementary Method S6. Multiplex immunofluorescence 5](#_Toc97212038)

[Supplementary Method S7. Peripheral blood samples and flow cytometry 6](#_Toc97212039)

[Supplementary Method S8. Assessment of tumor response and follow-up 6](#_Toc97212040)

[Supplementary Fig. S1. Work flow and patient selection. 7](#_Toc97212041)

[Supplementary Fig. S2. Association between the results of IHC, PCR, and NGS testing for identifying dMMR/MSI-H gastric adenocarcinoma. 8](#_Toc97212042)

[Supplementary Fig. S3. Mutations of PI3K-AKT-mTOR pathway of the concordant MSI-H cases in the surgery cohort. 9](#_Toc97212043)

[Supplementary Fig. S4. Representative images of hematoxylin-eosin and PD-L1 staining of MSI-H STAD samples in the 3DMed cohort. 10](#_Toc97212044)

[Supplementary Fig. S5. GSEA of gene signatures related to immune activation, interleukin pathways, and NOTCH signaling in comparisons between samples with or without mutation in PI3K-AKT-mTOR pathway. 11](#_Toc97212045)

[Supplementary Fig. S6. ROC curves illustrating the association of response rate with TMB and PD-L1 CPS. 12](#_Toc97212046)

[Supplementary Fig. S7. Predictive effect of PTEN mutation and NMP in *PTEN*^WT^ dMMR/MSI-H gastric adenocarcinomas. 13](#_Toc97212047)

[Supplementary Fig. S8. Association between mutations in PI3K-AKT-mTOR pathway and concentration of peripheral blood immune cells in patients with dMMR/MSI-H G/GEJ adenocarcinoma in the ICI treatment cohort. 14](#_Toc97212048)

[Supplementary Fig. S9. Prognostic effect of the genetic aberration in PI3K-AKT-mTOR pathway in the TCGA cohort. 15](#_Toc97212049)

[Supplementary Table S1. List of the genes in the 3DMed 733-gene panel. 16](#_Toc97212050)

[Supplementary Table S2. Members of the analyzed signaling pathways in the surgery cohort. 17](#_Toc97212051)

[Supplementary Table S3. Antibodies used in flow cytometry. 18](#_Toc97212052)

[Supplementary Table S4. List of gene signatures in GSEA. 19](#_Toc97212053)

[Supplementary Table S5. Clinicopathological and genomic characteristics in the surgery cohort. 22](#_Toc97212054)

[Supplementary Table S6. Association between the results of IHC, PCR, and NGS testing for identifying dMMR/MSI-H gastric adenocarcinoma. 24](#_Toc97212055)

[Supplementary Table S7 Genomic characteristics of the discordant samples. 25](#_Toc97212056)

[Supplementary Table S8. Clinicopathological characteristics of concordant dMMR/MSI-H samples according to the evaluation of immune infiltration in the surgery cohort. 26](#_Toc97212057)

[Supplementary Table S9. Clinicopathological characteristics of dMMR/MSI-H samples with evaluation of immune infiltration according to the history of neoadjuvant chemotherapy in the surgery cohort. 27](#_Toc97212058)

[Supplementary Table S10 Sensitivity analysis of the correlation between mutations in PI3K-AKT-mTOR pathway and immune cell infiltration. 28](#_Toc97212059)

[Supplementary Table S11. Detailed information of the MSI-H gastric adenocarcinoma samples evaluated by both 381-gene panel and PD-L1 kit retrieved from the 3DMed database. 29](#_Toc97212060)

[Supplementary Table S12. Clinicopathological characteristics of MSI-H STAD according to the genetic aberration in PI3K-AKT-mTOR pathway in the TCGA cohort 30](#_Toc97212061)

[Supplementary Table S13. The mutations of the members in the PI3K-AKT-mTOR pathway and mutation sites in the five MSI-H GAC cell lines 31](#_Toc97212062)

[Supplementary Table S14. The IC50 values of every cell line for the PI3K-AKT-mTOR inhibitors, the inhibitors targeting VEGFR or EGFR, and chemotherapeutic drugs 32](#_Toc97212063)

[Supplementary Table S15. Baseline characteristics of the patients with dMMR/MSI-H G/GEJ adenocarcinoma in the ICI treatment cohort. 33](#_Toc97212064)

[Supplementary Table S16. Univariable and multivariable analysis of PFS and OS in the ICI treatment cohort. 34](#_Toc97212065)

## Supplementary Method S1. Immunohistochemistry

Serial 5 μm thick sections from the formalin-fixed paraffin-embedded (FFPE) samples of primary G/GEJ tumors were cut onto glass slides for further IHC assessments. MMR proteins were assessed by a fully-automated staining device (Leica Bond-Ⅲ) with the following human-speciﬁc antibodies provided by Leica Biosystems Newcastle Ltd. (clone, dilution): MLH1 (ES05, 1:15, Gene Tech, South San Francisco, CA, USA), PMS2 (EP51, 1:30, Gene Tech), MSH2 (FE11, 1:150, Gene Tech), and MSH6 (EP49, 1:150, Gene Tech). “dMMR” was determined when there was a complete loss of at least one MMR protein. “Indeterminate” was determined when there was no complete loss of any MMR protein and an incomplete loss (geographic heterogeneity) of any MMR protein (for instance, intact MLH1/PMS2/MSH2 and partially lost MSH6). The immune cells in the central tumor area and invasive margin area were scored according to the guideline from the International Immuno-Oncology Biomarkers Working Group [20-21], labeled by antibodies including CD3 (LN10, 1:100, Leica), CD4 (UMAB64, 1:75, ORIGENE), CD8 (SP16, 1:50, BIOGENE), FOXP3 (236A/E7, 1:100, Abcam), and CD68 (KP1, 1:150, ZETA), performed as previously described [22]. All scoring was performed using ImageScope by two independent pathologists (X. Wang and Y. Sun), who were blinded to the results of NGS testing and clinical data. In addition, the detection of HER2 protein expression and chromogenic in-situ hybridization for EBV-encoded RNA (EBER) was performed as previously described [22]. PD-L1 IHC (Dako 22C3) was performed by the corresponding fully-automated staining device according to the manufacturer’s instructions.

## Supplementary Method S2. PCR testing of microsatellites

High-quality genomic DNA from FFPE tissues (including tumor and para-tumor tissue) were extracted using the QIAamp DNA FFPE Tissue Kit (Cat No. 56404, QIAGEN, Valencia, CA). Microsatellite analysis was assessed using the MSI analysis system (Lot No. X0270502211, MEDx (Suzhou) Translational Medicine Co., Ltd), composed of 9 pseudo-monomorphic mononucleotide repeats for detecting MSI, 2-pentanucleotide repeat loci (Penta C and Penta D) for confirming the identity between normal-source and tumor source samples, and 2-plasmid loci (QS90 and QS330) for quality control. In the present study, only the data of 5 traditional MSI loci (BAT26, NR24, NR21, MONO27, and BAT25) were evaluated. Cases were enrolled in this study when the proportion of tumor cells accounted for more than 30% of the total cells observed by microscopy, and the starting genomic DNA load was 10 ng, quantified with Qubit (FFPE samples). Para-tumor tissue was used as a negative control, and K562 cell line DNA was used as a positive control. PCR amplification was conducted on the SLAN-96P (Hongshitech, Shanghai, China), and then capillary electrophoresis was performed for fragment separation on ABI 3500Dx Genetic Analyzer (Invitrogen, Carlsbad, CA). Tumors were identified as MSI-H if two or more mononucleotide loci varied compared to the genomic DNA from normal tissue or blood. If only one mononucleotide loci varied, the sample was designated as MSI-L.

## Supplementary Method S3. NGS testing

The NGS testing of tumor DNA in FFPE samples and circulating tumor DNA (ctDNA) in plasma samples followed the method that had been previously reported [23-26], in a CAP- and CLIA-approved laboratory owned by the 3D Medicines Inc. The calling of variant by the 3DMed gene panel and the identification of MSI-H or microsatellite-stable (MSS) by the 3D-MSI panel were performed simultaneously, via the technique as previously published [27]. Germline mutations were filtered out using matched white blood cells before formal analysis of tumor genome sequencing data. The genomic features of 175 resected samples from patients with primary G/GEJ adenocarcinoma are drawn using R package ComplexHeatmap (version 2.9.3). The panels for tissue and plasma DNA testing are attached in **Supplementary Table S1**. The members of signaling pathways that were analyzed in the present study were determined according to The Cancer Genome Atlas (TCGA) database and our previous studies (**Supplemental Table S2**) [28].

## Supplementary Method S4. Gene set enrichment analysis (GSEA)

For GSEA [29], the javaGSEA Desktop Application (GSEA 4.0.1) was downloaded from http://software.broadinstitute.org/gsea/index.jsp. GSEA was used to associate the gene signature with genetic aberration in the PI3K-AKT-mTOR pathway and truncating *JAK1/2* mutation in MSI-H STAD. The signatures tested in the present study are shown in **Supplementary Table S3**. Fold-change values were exported for all genes and analyzed with version 4.0.1 of GSEA, using the GSEA pre-ranked module. The normalized enrichment score (NES) is the primary statistic for examining gene set enrichment results. The nominal P value estimates the statistical significance of the enrichment score. A gene set with nominal p<0.05 was determined to be significantly enriched in genes.

## Supplementary Method S5. Analysis of drug sensitivity of STAD cell lines

The data of STAD cell lines were retrieved from the cBioPortal (Cancer Cell Line Encyclopedia, Broad, 2019). Five of the 39 STAD cell lines were identified as MSI-H (NUGC-3, TGBC11TKB, SNU-1, IM95, and 23132/87). The IC_50_ was normalized by the natural logarithm of the ratio of individual IC_50_ to the geometric mean of IC_50_ for further comparisons.

## Supplementary Method S6. Multiplex immunofluorescence

Multiplex immunofluorescence staining was carried out using the Akoya OPAL Polaris 7-Color Automation IHC kit (NEL871001KT). FFPE tissue slides were incubated with specific primary antibodies targeting pan-CK (ab7753, 1:100, Abcam), CD3 (A0452, 1:100, Dako), CD4 (ab133616, 1:100, Abcam), CD8 (ab178089, 1:100, Abcam), CD56 (ab75813, 1:100, Abcam), CD68 (ab213363, 1:1000, Abcam), CD163 (ab182422, 1:500, Abcam), and FOXP3 (ab20034, 1:100, Abcam). This was followed by interaction with horseradish peroxidase-conjugated secondary antibody and tyramide signal amplification. The slides were heat-treated after each round of amplification. Cell nuclei acids were counterstained with 4’, 6-diamidino-2-phenylindole (DAPI, SIGMA-ALDRICH). Multiplex-stained slides were scanned, and all scans for each slide were then merged to determine the relative localizations of the proteins. The quantities of various cell populations were expressed as the number of stained cells per square millimeter. A comprehensive correlation matrix was created by R package corrplot (version 0.92) to seek the genomic correlates of the density of tumor-infiltrating immune cells.

## Supplementary Method S7. Peripheral blood samples and flow cytometry

White blood cell counts, lymphocyte counts, and lymphocyte subset counts were obtained from the peripheral blood within 3 days before the initial immunotherapy. Lymphocyte subset counts were obtained by flow cytometry in the clinical laboratory of Peking University Cancer Hospital & Institute. Whole blood was collected aseptically by venipuncture, using BD Vacutainer EDTA blood collection tubes. A minimum of 50 μL of whole blood is required for this procedure. Anticoagulated blood stored at room temperature (20℃–25℃) must be stained within 24 hours of draw and must be analyzed within 6 hours of staining. Cells were incubated for 15 minutes in the dark at room temperature (20℃–25℃), with fluorophore-conjugated antibodies as indicated in **Supplemental Table S4**. After hemolysis 10 min and gradients centrifugation, the cells were washed twice in PBS, and then analyzed with BD FACSCanto II.

There were seven subsets evaluated in the lymphocyte subset counts: CD3+ T cells, CD4+ T cells (CD3^+^/CD4^+^), CD8^+^ T cells (CD3^+^/CD8^+^), suppressor T (Ts) cells (CD3^+^/CD8^+^/CD28^-^), cytotoxic T (Tc) cells (CD3^+^/CD8^+^/CD28^+^), natural killer (NK) cells (CD3^-^/CD16^+^/CD56^+^), and regulatory T (Treg) cells (CD3^+^/CD4^+^/CD25^high^/CD127^low^).

## Supplementary Method S8. Assessment of tumor response and follow-up

In the ICI cohort, tumor assessment was implemented every 4-6 weeks by two independent investigators (Z. Wang, and L. Shen) blinded from the data of NGS testing. ORR was defined as the percentage of patients with confirmed complete response (CR) or partial response (PR) by Response Evaluation Criteria in Solid Tumours v1.1 (RECIST v1.1). Progression-free survival (PFS) was defined as the time from the start of immunotherapy until disease progression or death from any cause. Overall survival (OS) was defined as the time from the first dose to the date of last known contact or death.

## Supplementary Fig. S1. Work flow and patient selection.

**
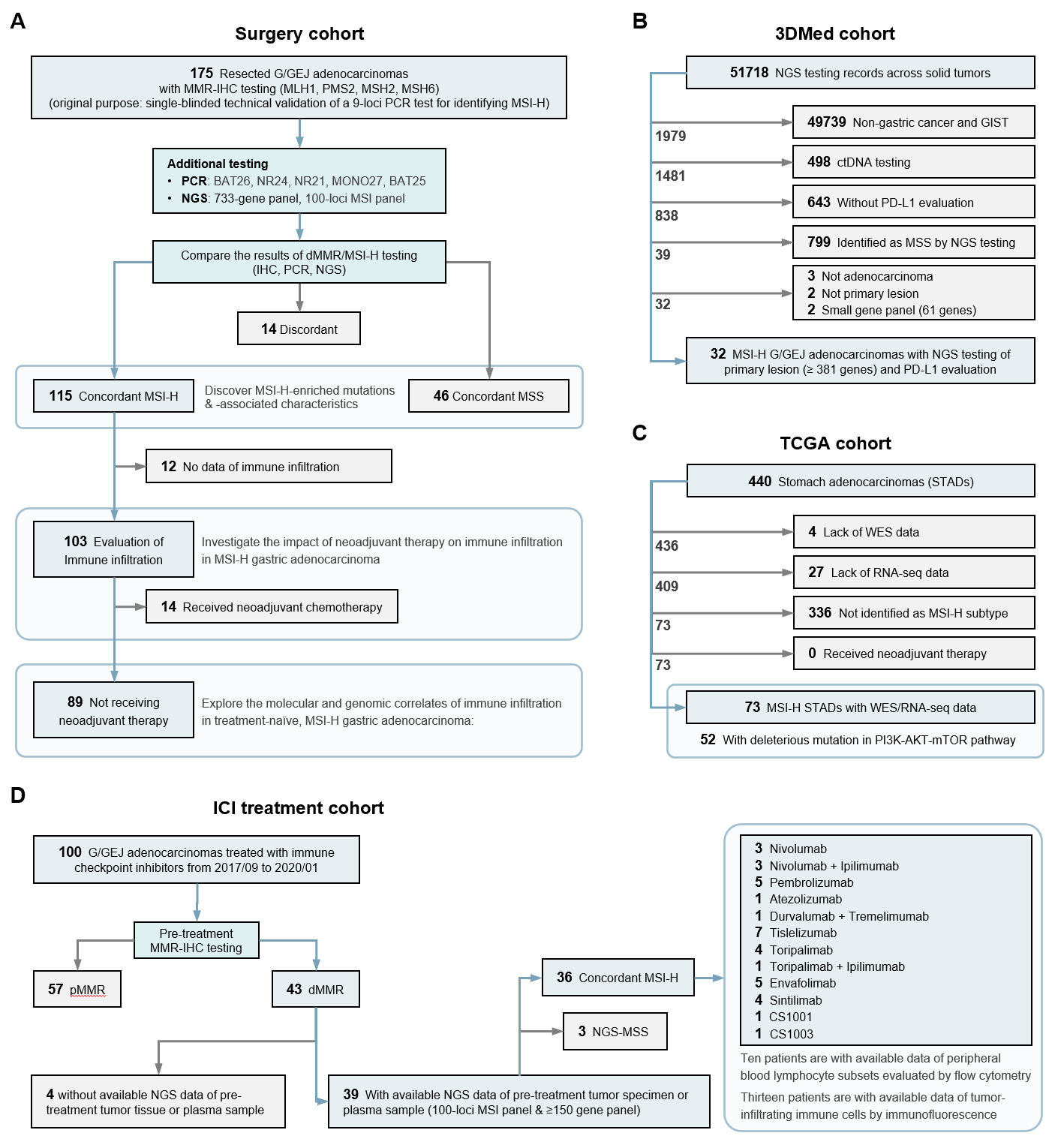
**

**A-D**. The work flow and patient selection of the surgery cohort (**A**), the 3DMed cohort (**B**), the TCGA cohort (**C**), and the ICI treatment cohort (**D**). Abbreviations: dMMR=mismatch repair-deficient, G/GEJ=gastric/gastroesophageal junction, GIST=gastrointestinal stromal tumor, ICI=immune checkpoint inhibitor, IHC=immunohistochemistry, MLH1=MutL homolog 1, MMR=mismatch repair, MSH2=MutS Homolog 2, MSH6=MutS Homolog 6, MSI=microsatellite instability, MSI-H=microsatellite instability-high, MSS=microsatellite stability, NAC=neoadjuvant chemotherapy, NGS=next-generation sequencing, PCR=polymerase chain reaction, PD-L1=programmed death-ligand 1, pMMR=mismatch repair-proficient, PMS2=PMS1 Homolog 2, STAD=stomach adenocarcinoma, TCGA=The Cancer Genome Atlas, WES=whole-exome sequencing.

## Supplementary Fig. S2. Association between the results of IHC, PCR, and NGS testing for identifying dMMR/MSI-H gastric adenocarcinoma.

X axis represents MSI score (cut-off for MSI-H is 30). Y axis represents PCR score (cut-off for MSI-H is 2). Every point represents a sample, and its color reflects the MMR protein status according to the legend at the upper left corner. Abbreviations: dMMR=mismatch repair-deficient, IHC=immunohistochemistry, MSI=microsatellite instability, PCR=polymerase chain reaction, pMMR=mismatch repair-proficient.

## Supplementary Fig. S3. Mutations of PI3K-AKT-mTOR pathway of the concordant MSI-H cases in the surgery cohort.


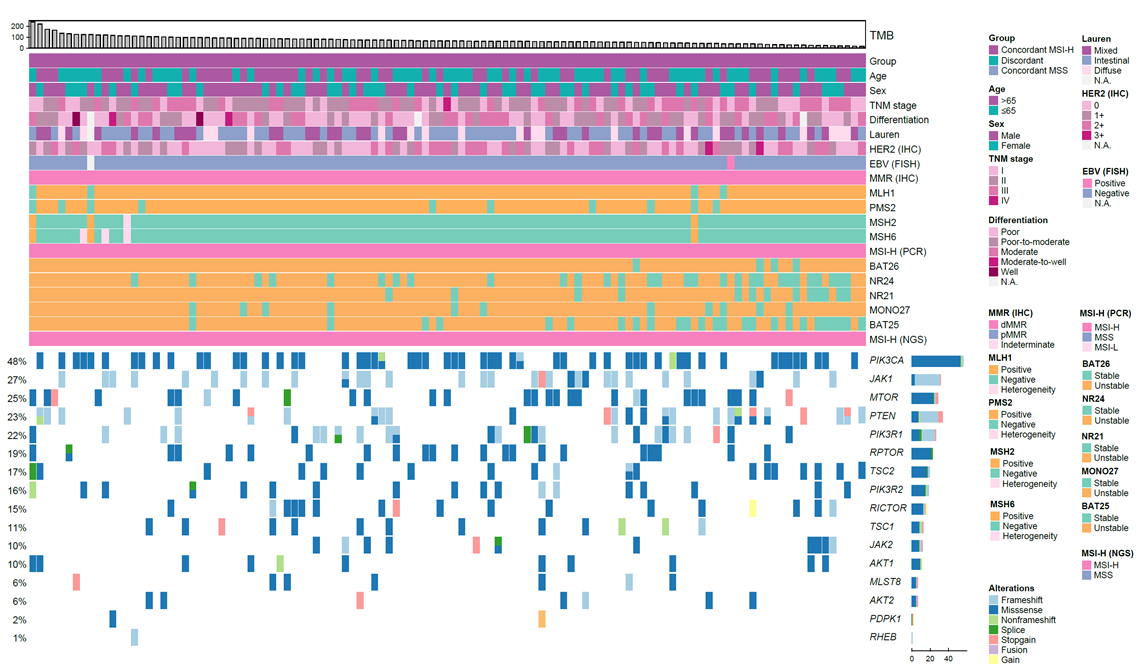


Tumor mutational burden is shown in the upper panel. Basic clinicopathological characteristics are illustrated in the middle panel. Mutations of members of PI3K-AKT-mTOR pathway and *JAK1/2* are depicted in the lower panel. Abbreviations: dMMR=mismatch repair-deficient, EBV=Epstein-Barr virus, FISH=fluorescence in situ hybridization, HER2=human epidermal growth factor receptor 2, IHC=immunohistochemistry, MLH1=MutL homolog 1, MMR=mismatch repair, MSH2=MutS Homolog 2, MSH6=MutS Homolog 6, MSI-H=microsatellite instability-high, MSI-L=microsatellite instability-low, MSS=microsatellite stability, N.A.=not applicable, NGS=next-generation sequencing, PCR=polymerase chain reaction, PMS2=PMS1 Homolog 2, TMB=tumor mutational burden.

## Supplementary Fig. S4. Representative images of hematoxylin-eosin and PD-L1 staining of MSI-H STAD samples in the 3DMed cohort.


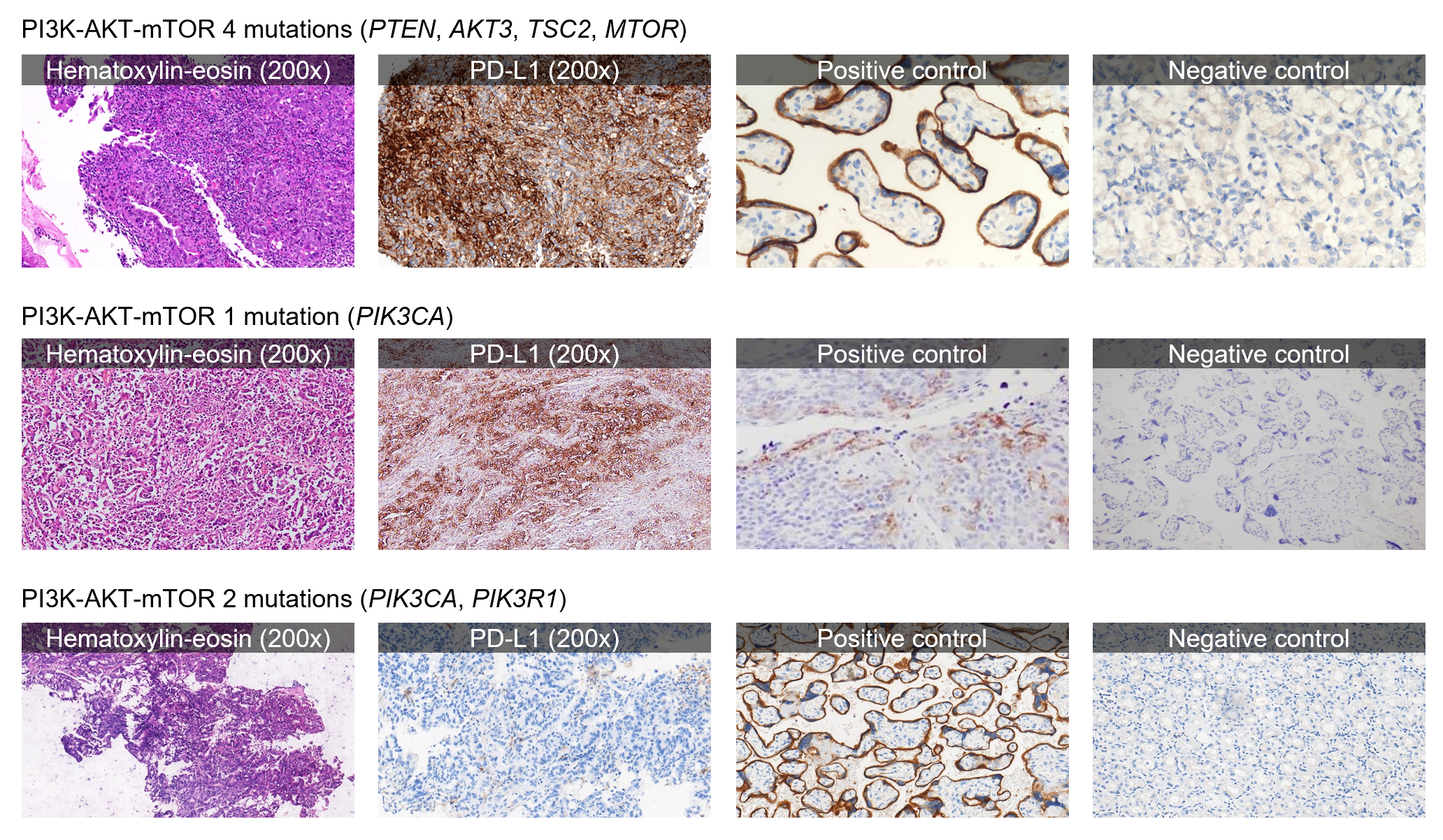


Abbreviations: MSI-H=microsatellite instability-high, PD-L1=programmed death-ligand 1, STAD=stomach adenocarcinoma.

## Supplementary Fig. S5. GSEA of gene signatures related to immune activation, interleukin pathways, and NOTCH signaling in comparisons between samples with or without mutation in PI3K-AKT-mTOR pathway.


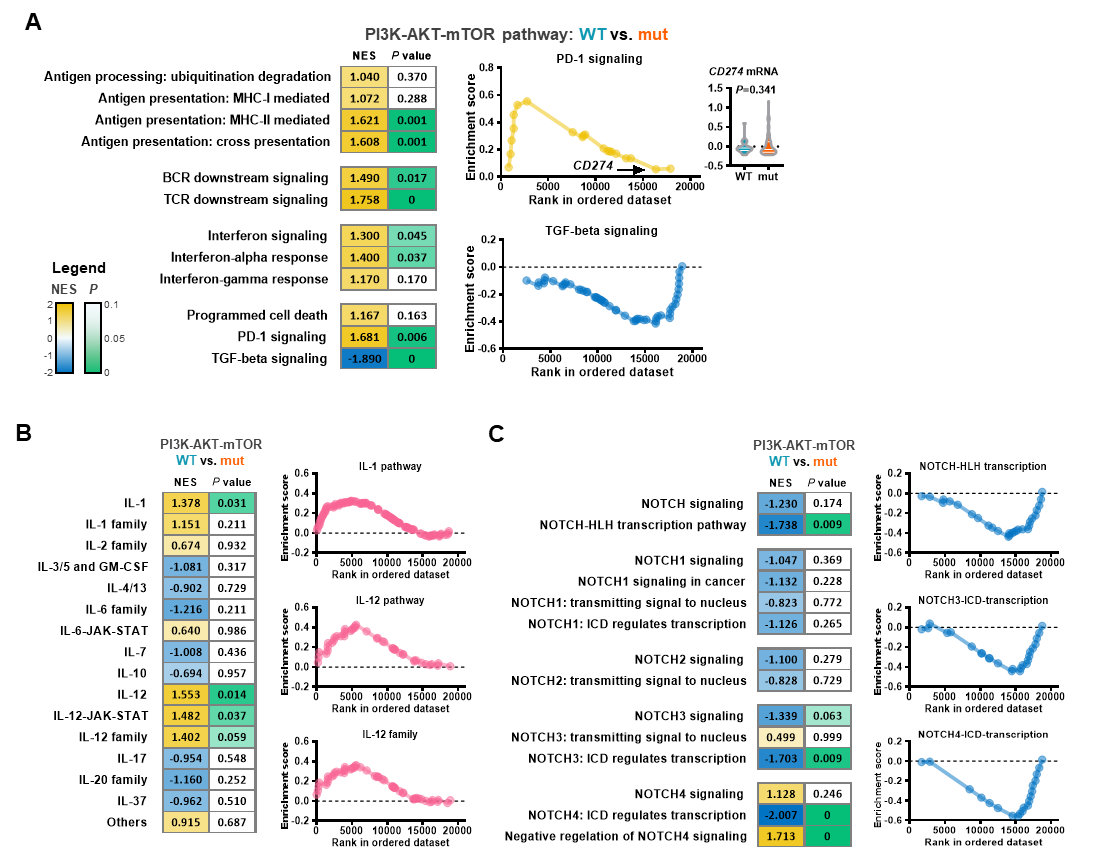


**A**. GSEA of gene signatures related to immune activation in comparisons between samples with or without mutation in PI3K-AKT-mTOR pathway. The yellow-blue scale represents NES. The green scale represents P value. The plots of enrichment scores of PD-1 signaling, and TGF-β signaling, together with the comparisons of PD-L1 (CD274) mRNA expression. **B-C**. GSEA of gene signatures related to interleukin pathways (**B**), and NOTCH signaling (**C**) in comparisons between samples with or without mutation in PI3K-AKT-mTOR pathway. The yellow-blue scale represents NES. The green scale represents P value. Abbreviations: BCR=B cell receptor, IL=interleukin, MHC=major histocompatibility complex, NES=normalized enrichment score, PD-1=programmed death-1, PD-L1=programmed death-ligand 1, TCR=T cell receptor, TGF=transforming growth factor, WT=wildtype.

## Supplementary Fig. S6. ROC curves illustrating the association of response rate with TMB and PD-L1 CPS.


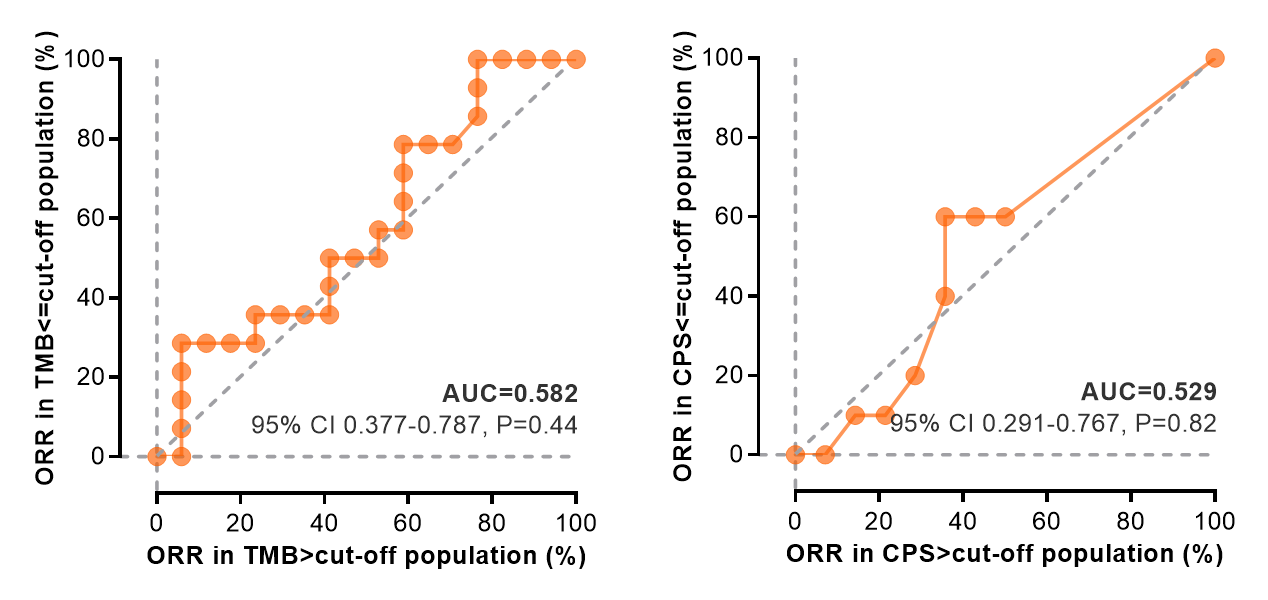


ORR-based ROC for TMB and PD-L1 CPS. Abbreviations: CPS=combined positive score, ORR=objective response rate, PD-L1=programmed death-ligand 1, ROC=receiver operator characteristic, TMB=tumor mutational burden.

## Supplementary Fig. S7. Predictive effect of PTEN mutation and NMP in *PTEN*^WT^ dMMR/MSI-H gastric adenocarcinomas.


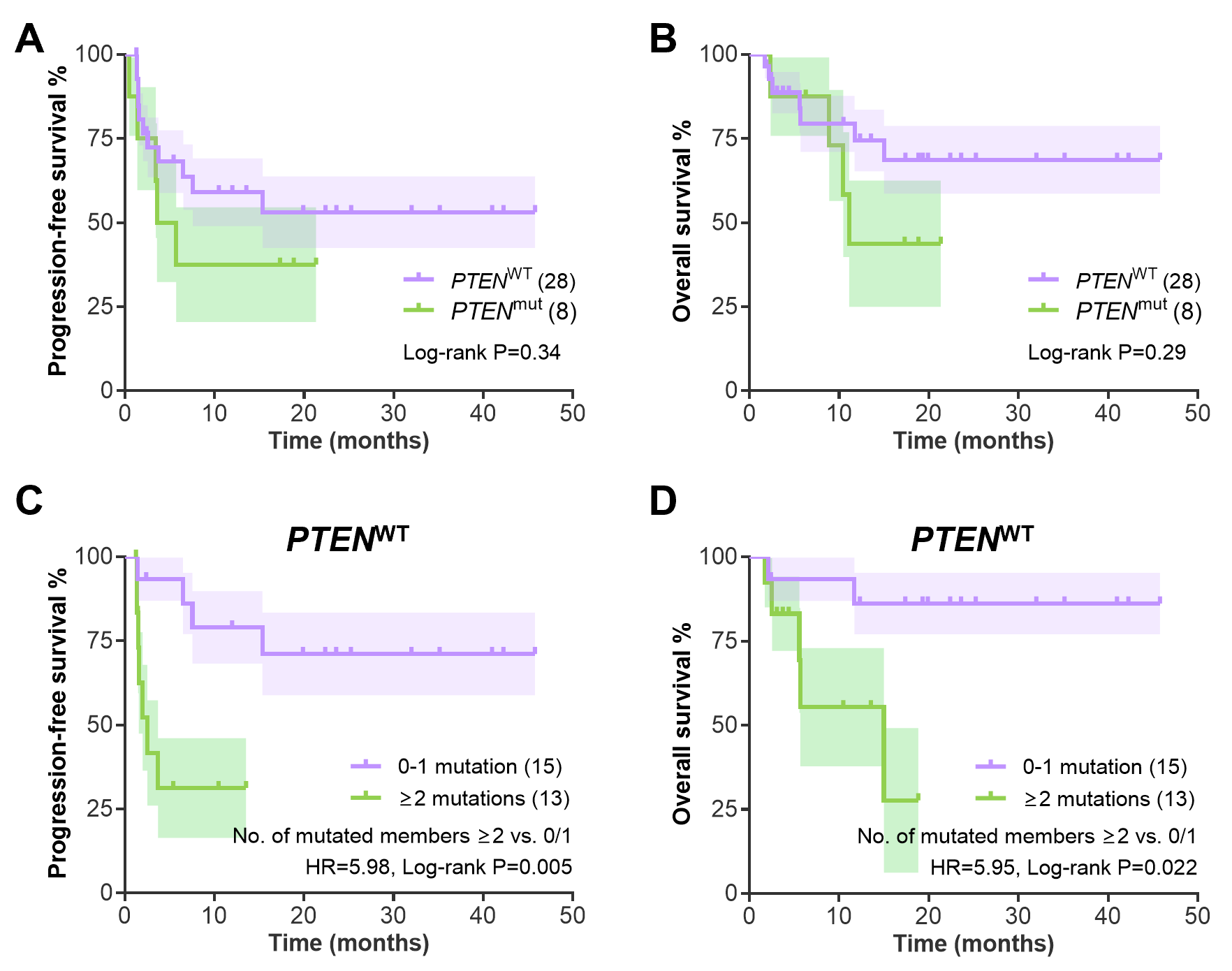


**A-B**. Kaplan-Meier curves of the PFS (**A**) and OS (**B**) of the patients in the ICI treatment cohort grouped by *PTEN* mutation. **C-D**. Kaplan-Meier curves of the PFS (**C**) and OS (**D**) of the *PTEN*^WT^ patients in the ICI treatment cohort grouped by NMP. Abbreviations: NMP=number of mutated genes in the PI3K-AKT-mTOR pathway, OS=overall survival, PFS=progression-free survival.

##
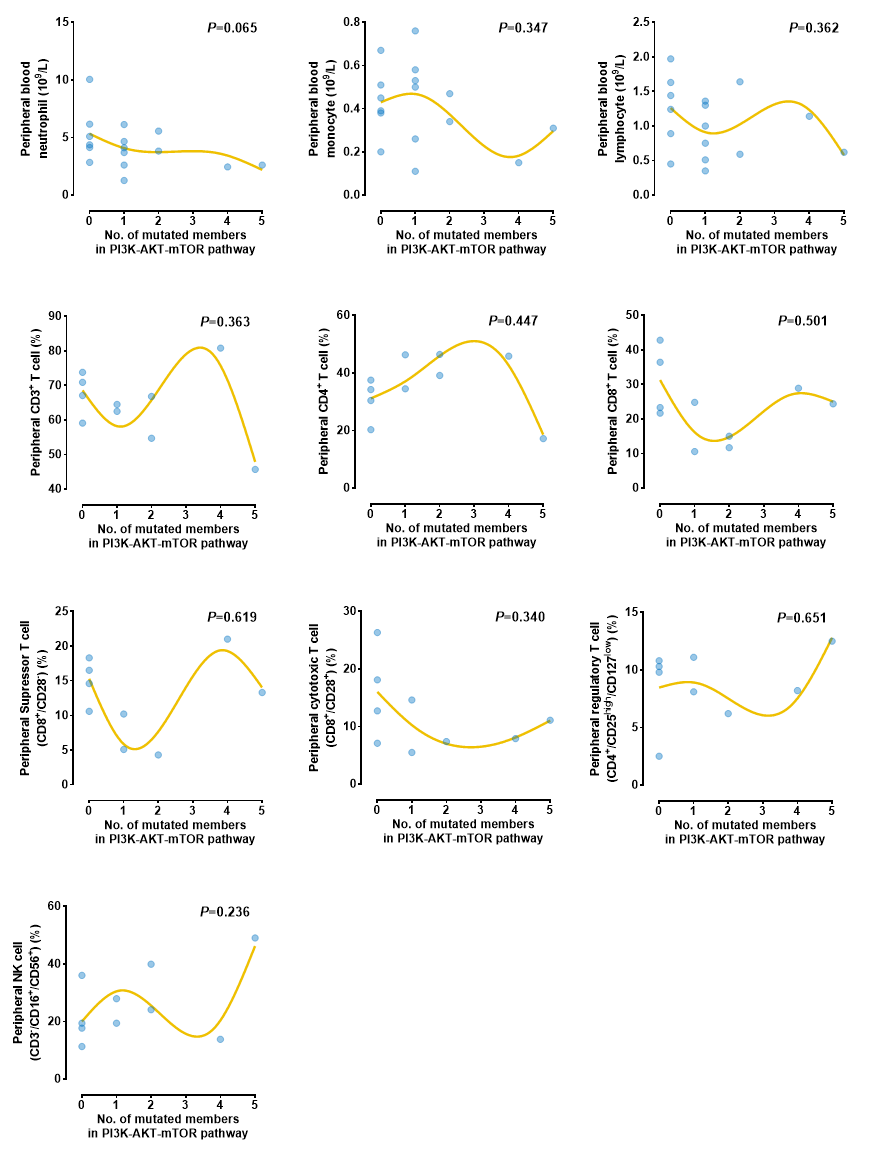
Supplementary Fig. S8. Association between mutations in PI3K-AKT-mTOR pathway and concentration of peripheral blood immune cells in patients with dMMR/MSI-H G/GEJ adenocarcinoma in the ICI treatment cohort.

Non-parametric correlation between the mutated member of PI3K-AKT-mTOR pathway and the peripheral blood immune cells before the initiation of ICI treatment, evaluated by blood routine examination or flow cytometry of the pretreatment peripheral blood. Abbreviations: dMMR=mismatch repair-deficient, G/GEJ=gastric/gastroesophageal junction, ICI=immune checkpoint inhibitor, MSI-H=microsatellite instability-high.

## Supplementary Fig. S9. Prognostic effect of the genetic aberration in PI3K-AKT-mTOR pathway in the TCGA cohort.


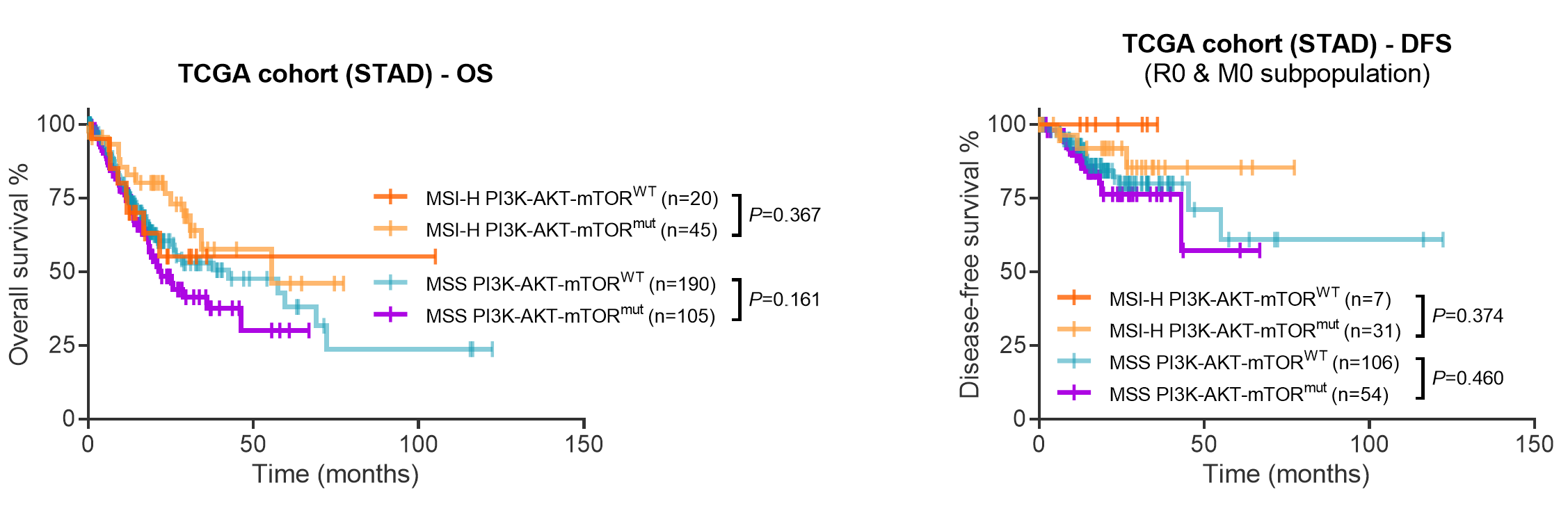


Kaplan-Meier curves of the OS and DFS of the STAD patients grouped by the genetic aberration in PI3K-AKT-mTOR pathway. Abbreviations: DFS=disease-free survival, TCGA=The Cancer Genome Atlas, OS=overall survival.

## Supplementary Table S1. List of the genes in the 3DMed 733-gene panel.

| **Gene list** |
| --- |
| *ABCB11 ABI1 ABL1 ABRAXAS1 ACKR3 ACSL3 ACVR1 ACVR1B ACVR2A AEN AFF3 AFF4 AKT1 AKT2 AKT3 ALK ALKBH2 ALKBH3 AMER1 ANK1 APC APEX1 APEX2 APLF APOBEC3B APTX AR ARAF AREG ARHGAP5 ARID1A ARID1B ARID2 ARNT ASXL1 ATM ATP1A1 ATP2B3 ATR ATRIP ATRX AURKA AXIN1 AXIN2 AXL B2M BAP1 BARD1 BAZ1A BCL10 BCL11A BCL11B BCL2 BCL2L1 BCL2L11 BCL6 BCOR BCORL1 BCR BIRC3 BIRC5 BLM BMP5 BMPR1A BRAF BRCA1 BRCA2 BRD4 BRIP1 BTG1 BTK BUB1B CACNA1D CALR CAMTA1 CANT1 CARD11 CARS CASP8 CBFA2T3 CBFB CBL CBLB CCDC6 CCNB1IP1 CCND1 CCND2 CCND3 CCNE1 CCNH CCNO CD274 CD74 CD79A CD79B CDC73 CDH1 CDH10 CDH11 CDK12 CDK2 CDK4 CDK6 CDK7 CDK8 CDKN1A CDKN1B CDKN1C CDKN2A CDKN2B CDKN2C CDX2 CEBPA CENPS CENPX CETN2 CHAF1A CHD1 CHD2 CHD4 CHEK1 CHEK2 CHIC2 CIC CIITA CLIP1 CLK2 CLTCL1 CNBP CNOT3 COL7A1 CRBN CREB3L1 CREB3L2 CREBBP CRKL CRLF2 CRNKL1 CRTC1 CRTC3 CSF1R CSF3R CTCF CTNNB1 CTNND2 CTR9 CUL1 CUL3 CUL4A CUL5 CUX1 CXCR4 CYLD CYP17A1 CYP2C19 CYP2D6 CYSLTR2 DAXX DCLRE1A DCLRE1B DCLRE1C DDB1 DDB2 DDIT3 DDR2 DDX10 DDX3X DDX5 DDX6 DICER1 DIS3 DIS3L2 DKC1 DMC1 DNM2 DNMT1 DNMT3A DNTT DOCK8 DPYD DROSHA DUT EBF1 EED EGFR EIF3E EIF4A2 ELANE ELF3 ELF4 ELK4 ELL ELOA EME1 EME2 EMSY ENDOV EP300 EPAS1 EPCAM EPHA2 EPHA3 EPHA7 EPHB1 EPS15 ERBB2 ERBB3 ERBB4 ERC1 ERCC1 ERCC2 ERCC3 ERCC4 ERCC5 ERCC6 ERCC8 EREG ERF ERRFI1 ESR1 ETNK1 ETV1 ETV4 ETV5 ETV6 EWSR1 EXO1 EXT1 EXT2 EZH2 EZR FAAP100 FAAP20 FAAP24 FAH FAM135B FAM47C FAN1 FANCA FANCB FANCC FANCD2 FANCE FANCF FANCG FANCI FANCL FANCM FAS FAT1 FAT4 FBXW7 FEN1 FES FGF19 FGF3 FGF4 FGFR1 FGFR2 FGFR3 FGFR4 FH FHIT FLCN FLT1 FLT3 FLT4 FOXA1 FOXL2 FOXP1 FRK FRS2 FUBP1 FUS G6PD GALNT12 GAS7 GATA1 GATA2 GATA3 GBA GEN1 GFI1 GJB2 GLI1 GLI2 GLI3 GNA11 GNA13 GNAQ GNAS GPC3 GRB2 GREM1 GRIN2A GSK3B GSTT1 GTF2H1 GTF2H3 GTF2H4 GTF2H5 H2AFX H3F3A HDAC1 HDAC2 HELQ HES1 HEY1 HFE HFM1 HGF HIF1A HIP1 HIST1H3B HLTF HMBS HMGA2 HMGB1 HNF1A HNRNPA2B1 HOOK3 HOXA11 HOXB13 HRAS HUS1 HUS1B IDH1 IDH2 IGF1R IGF2 IKBKE IKZF1 IL6ST IL7R INPP4B IRS2 ITGAV ITK JAK1 JAK2 JAK3 JMJD1C JUN KCNJ5 KDM5A KDM5C KDM6A KDR KEAP1 KIT KLF4 KMT2A KMT2C KMT2D KNL1 KRAS LASP1 LATS1 LATS2 LCK LEF1 LIFR LIG1 LIG3 LIG4 LMNA LMO1 LRP1B LZTR1 MAD2L2 MAP2K1 MAP2K2 MAP2K4 MAP3K1 MAPK1 MAX MBD4 MCL1 MDC1 MDM2 MDM4 MECOM MED12 MEF2B MEN1 MET MGA MGMT MITF MLH1 MLH3 MLLT3 MLST8 MMS19 MNAT1 MPG MPL MPLKIP MRE11 MSH2 MSH3 MSH4 MSH5 MSH6 MTAP MTOR MUS81 MUTYH MYB MYC MYCL MYCN MYD88 MYOD1 NAB2 NABP2 NBN NCOA3 NCOR1 NCOR2 NDRG1 NEIL1 NEIL2 NEIL3 NF1 NF2 NFE2L2 NFIB NFKBIA NHEJ1 NHP2 NKX2-1 NME1 NONO NOP10 NOTCH1 NOTCH2 NOTCH3 NOTCH4 NPM1 NR4A3 NRAS NRG1 NRG3 NSD2 NSD3 NT5C2 NTHL1 NTRK1 NTRK2 NTRK3 NUDT1 NUP93 NUTM1 OGG1 PAK1 PALB2 PARP1 PARP2 PARP3 PARP4 PAX3 PAX5 PAX7 PAX8 PBRM1 PCDH9 PCNA PDCD1LG2 PDGFB PDGFRA PDGFRB PDPK1 PER1 PER2 PER3 PHF6 PHOX2B PICALM PIK3CA PIK3CB PIK3CD PIK3R1 PIK3R2 PIK3R3 PIM1 PLCG2 PLXNA1 PLXNB1 PML PMS1 PMS2 PNKP POLB POLD1 POLD3 POLD4 POLE POLE2 POLE3 POLE4 POLG POLH POLI POLK POLL POLM POLN POLQ POT1 POU2AF1 POU5F1 PPARG PPM1D PPP2R1A PPP2R2A PPP4R1 PPP4R2 PPP4R3A PPP4R3B PPP4R4 PPP6C PRCC PRDM1 PRDM16 PRDM9 PREX2 PRF1 PRKACA PRKAR1A PRKCH PRKDC PRPF19 PRSS1 PSIP1 PTCH1 PTEN PTK2 PTK6 PTPN11 PTPN13 PTPRD PTPRT QKI RAC1 RAD1 RAD18 RAD21 RAD23A RAD23B RAD50 RAD51 RAD51B RAD51C RAD51D RAD52 RAD54B RAD54L RAD54L2 RAD9A RAD9B RAF1 RANBP2 RAP1GDS1 RARA RASA1 RB1 RBBP8 RBM10 RBX1 RDM1 RECQL RECQL4 RECQL5 RET REV1 REV3L RFC1 RFC2 RFC3 RFC4 RFC5 RFWD3 RGS7 RHBDF2 RHEB RHOA RHOH RICTOR RIF1 RIT1 RMI1 RMI2 RNF168 RNF213 RNF4 RNF43 RNF8 ROS1 RPA1 RPA2 RPA3 RPA4 RPS6KA3 RPS6KB1 RPTOR RRM2B RUNX1 RUNX1T1 RXRA SBDS SDC4 SDHA SDHAF2 SDHB SDHC SDHD SEM1 SERPINA1 SERPINB3 SETBP1 SETD2 SETMAR SF3B1 SFPQ SGK1 SH2B3 SH2D1A SHOC2 SHPRH SLC25A13 SLC29A1 SLC34A2 SLC45A3 SLIT2 SLX1A SLX4 SMAD2 SMAD3 SMAD4 SMARCA1 SMARCA2 SMARCA4 SMARCB1 SMO SMUG1 SOCS1 SOS1 SOX2 SOX9 SPEN SPO11 SPOP SPRED1 SPRTN SPTA1 SRC SRGAP3 SRSF2 SRY SS18 STAG2 STAT3 STK11 SUFU SUZ12 SYK TBL1XR1 TBX3 TCF3 TCF7L2 TCL1A TDG TDP1 TDP2 TEAD2 TELO2 TERT TET1 TET2 TFE3 TGFBR1 TGFBR2 THBS2 TIMELESS TMEM127 TMEM189 TMPRSS2 TNFAIP3 TOP2A TOP3A TOP3B TOPBP1 TP53 TP53BP1 TP63 TPMT TRAF7 TREX1 TREX2 TRIM37 TSC1 TSC2 TSHR TSPAN31 TYK2 U2AF1 UBE2A UBE2B UBE2N UBE2T UBE2V2 UGT1A1 UNG UROD USP1 USP6 USP8 UVSSA VEGFA VHL WAS WDR48 WIF1 WRN WT1 XAB2 XPA XPC XPO1 XRCC1 XRCC2 XRCC3 XRCC4 XRCC5 XRCC6 YAP1 YWHAE ZBTB16 ZFHX3 ZNF217 ZNF479 ZNF703 ZNF750 ZNRF3* |

## Supplementary Table S2. Members of the analyzed signaling pathways in the surgery cohort.

| **Pathway** | **Gene list** |
| --- | --- |
| Cell cycle | *RB1 CCNE1 CDK2 CCND1 CDK4 CDK6 CCND2 CDKN2A CDKN2B MYC CDKN1A CDKN1B SRC JAK1 JAK2 STAT3* |
| p53 gene | *TP53 MDM2 MDM4 CDKN2A CDKN2B TP53BP1* |
| Notch signaling | *CUL1 EP300 FBXW7 HDAC1 HDAC2 HES1 KDM5A NCOR2 NOTCH1 NOTCH2 NOTCH3 NOTCH4 SPEN HEY1* |
| PI3K-AKT-mTOR pathway | *PIK3CA PIK3R1 PIK3R2 PTEN PDPK1 AKT1 AKT2 MTOR RICTOR TSC1 TSC2 RHEB RPTOR MLST8* |
| RTK signaling | *EGFR ERBB2 ERBB3 ERBB4 PDGFB PDGFRA PDGFRB KIT FGFR1 IGF1R VEGFA KDR* |
| Ras-Raf-MEK-Erk/JNK pathway | *KRAS HRAS BRAF RAF1 MAP3K1 MAP2K1 MAP2K2 MAP2K4 MAPK1* |
| TGF-beta signaling | *TGFBR1 TGFBR2 BMP5 BMPR1A ACVR1 ACVR1B ACVR2A SMAD2 SMAD3 SMAD4* |
| WNT signaling | *AMER1 APC AXIN1 AXIN2 CDC73 CTNNB1 FOXP1 GREM1 GSK3B KMT2D LRP1B PAX5 RNF43 SLIT2 SOX2 SOX9 TERT ZNRF3* |
| Telomere maintenance | *TERT* |
| Hippo pathway | *FAT1 FAT4 NF1 NF2 LATS1 LATS2 TEAD2* |
| Hedgehog pathway | *SMO GLI1 GLI2 GLI3* |
| Chromatin remodeling | *ARID1A ARID1B ARID2 PBRM1 SMARCA1 SMARCA2 SMARCA4 SMARCB1 EZH2 SETD2 KMT2A KMT2D KMT2C* |
| Cell adhesion | *FAT1 FAT4 RHOA CDH1* |
| Base excision repair | *APEX1 APLF APTX CCNO FEN1 HMGB1 LIG1 LIG3 MBD4 MPG MUTYH NEIL1 NEIL2 NEIL3 NTHL1 OGG1 PARP1 PARP2 PARP3 PARP4 PCNA PNKP POLE POLB POLD1 POLD3 POLD4 POLE2 POLE3 POLE4 POLL SMUG1 TDG TDP1 UNG XRCC1* |
| Checkpoint factors | *AEN ATM ATR ATRIP CHEK1 CHEK2 HUS1 HUS1B PER1 PER2 PER3 RAD1 RAD9A RAD9B RFC2 RFC3 RFC4 RFC5 TIMELESS TP53* |
| Fanconi anemia pathway | *CENPS BLM BRCA1 BRCA2 BRIP1 FAAP100 FAAP24 FAN1 FANCA FANCC FANCD2 FANCE FANCF FANCG FANCI FANCL FANCM HES1 PALB2 RAD51 RAD51C RMI1 RMI2 CENPX TELO2 TOP3A TOP3B UBE2T USP1 WDR48* |
| Homologous recombination repair | *BLM BRCA1 BRCA2 DMC1 EME1 EME2 GEN1 HFM1 MRE11 MUS81 NBN PPP4R1 PPP4R2 PPP4R4 RAD50 RAD51 RAD51B RAD51C RAD51D RAD52 RAD54B RAD54L RAD54L2 RDM1 RECQL RECQL4 RECQL5 RMI1 RMI2 RPA1 RPA2 RPA3 SEM1 SLX1A SLX4 PPP4R3A PPP4R3B SPO11 TOP3A TOP3B WRN XRCC2 XRCC3* |
| Mismatch repair | *EXO1 HMGB1 LIG1 MLH1 MLH3 MSH2 MSH3 MSH4 MSH5 MSH6 PCNA PMS1 PMS2 POLD1 POLD3 POLD4 RFC1 RFC2 RFC3 RFC4 RFC5 RPA1 RPA2 RPA3* |
| Nucleotide excision repair | *CCNH CDK7 CUL3 CUL4A CUL5 DDB1 DDB2 ERCC1 ERCC2 ERCC3 ERCC4 ERCC6 ERCC8 GTF2H1 GTF2H3 GTF2H4 GTF2H5 LIG1 MMS19 MNAT1 RAD23A RAD23B RBX1 RPA1 RPA2 RPA3 ELOA XPA XPC* |
| Nonhomologous end-joining | *APLF APTX DCLRE1C DNTT LIG4 NHEJ1 POLB POLL POLM PRKDC RAD50 XRCC4 XRCC5 XRCC6* |
| Translesion DNA synthesis | *HLTF POLH POLI POLK POLN RAD18 REV1 REV3L TMEM189 UBE2B UBE2N UBE2V2* |

## Supplementary Table S3. Antibodies used in flow cytometry.

| Evaluated subset | Target | Fluorophore | Clone | Manufacturer |
| --- | --- | --- | --- | --- |
| CD3+ T cells (CD3^+^),  CD4+ T cells (CD3^+^/CD4^+^),  CD8^+^ T cells (CD3^+^/CD8^+^),  Natural killer cells (CD3^-^/CD16^+^/CD56^+^) | CD3 | FITC | SK7 | BD Biosciences |
|  | CD4 | PE-Cy7 | SK3 |  |
|  | CD8 | APC-Cy7 | SK1 |  |
|  | CD56 | PE | NCAM16.2 |  |
| Regulatory T cells  (CD3^+^/CD4^+^/CD25^high^/CD127^low^) | CD3 | PerCP | SK7 |  |
|  | CD127 | Alexa Fluor 647 | HIL-7R-M21 |  |
|  | CD25 | PE | 2A3 |  |
|  | CD4 | FITC | SK3 |  |
| Suppressor T (Ts) cells (CD3^+^/CD8^+^/CD28^-^),  Cytotoxic T (Tc) cells (CD3^+^/CD8^+^/CD28^+^) | CD3 | PerCP | SK7 |  |
|  | CD8 | APC-Cy7 | SK1 |  |
|  | CD28 | PE | L293 |  |

## Supplementary Table S4. List of gene signatures in GSEA.

| Function | Detailed function | Original size | Size after restricting to dataset | Name of gene signature |
| --- | --- | --- | --- | --- |
| DNA damage response | DNA repair | 331 | 301 | [REACTOME_DNA_REPAIR](http://www.gsea-msigdb.org/gsea/msigdb/cards/REACTOME_DNA_REPAIR) |
|  | Homologous recombination repair | 67 | 58 | REACTOME_HDR_THROUGH_HOMOLOGOUS_RECOMBINATION_HRR |
|  | Mismatch repair | 15 | 15 | [REACTOME_MISMATCH_REPAIR](http://www.gsea-msigdb.org/gsea/msigdb/cards/REACTOME_MISMATCH_REPAIR) |
|  | Base excision repair | 91 | 85 | [REACTOME_BASE_EXCISION_REPAIR](http://www.gsea-msigdb.org/gsea/msigdb/cards/REACTOME_BASE_EXCISION_REPAIR) |
|  | Nucleotide excision repair | 110 | 109 | [REACTOME_NUCLEOTIDE_EXCISION_REPAIR](http://www.gsea-msigdb.org/gsea/msigdb/cards/REACTOME_NUCLEOTIDE_EXCISION_REPAIR) |
|  | Fanconi anemia pathway | 39 | 30 | [REACTOME_FANCONI_ANEMIA_PATHWAY](http://www.gsea-msigdb.org/gsea/msigdb/cards/REACTOME_FANCONI_ANEMIA_PATHWAY) |
|  | Translesion DNA synthesis | 39 | 36 | REACTOME_TRANSLESION_SYNTHESIS_BY_Y_FAMILY_DNA_POLYMERASES_BYPASSES_LESIONS_ON_DNA_TEMPLATE |
|  | Non-homologous end-joining | 69 | 60 | REACTOME_NONHOMOLOGOUS_END_JOINING_NHEJ |
|  | Checkpoint factors | 95 | 84 | REACTOME_G2_M_DNA_DAMAGE_CHECKPOINT |
| Antigen processing | Antigen processing: ubiquitination degradation | 308 | 288 | [REACTOME_ANTIGEN_PROCESSING_UBIQUITINATION_PROTEASOME_DEGRADATION](http://www.gsea-msigdb.org/gsea/msigdb/cards/REACTOME_ANTIGEN_PROCESSING:_UBIQUITINATION_PROTEASOME_DEGRADATION) |
| Antigen presentation | Antigen presentation: MHC-I-mediated | 350 | 350 | [REACTOME_CLASS_I_MHC_MEDIATED_ANTIGEN_PROCESSING_PRESENTATION](http://www.gsea-msigdb.org/gsea/msigdb/cards/REACTOME_CLASS_I_MHC_MEDIATED_ANTIGEN_PROCESSING_PRESENTATION) |
|  | Antigen presentation: MHC-II-mediated | 124 | 117 | REACTOME_MHC_CLASS_II_ANTIGEN_PRESENTATION |
|  | Antigen presentation: cross presentation | 99 | 95 | [REACTOME_ANTIGEN_PROCESSING_CROSS_PRESENTATION](http://www.gsea-msigdb.org/gsea/msigdb/cards/REACTOME_ANTIGEN_PROCESSING_CROSS_PRESENTATION) |
| BCR/TCR signaling | BCR downstream signaling | 81 | 80 | [REACTOME_](http://www.gsea-msigdb.org/gsea/msigdb/cards/REACTOME_SIGNALING_BY_THE_B_CELL_RECEPTOR_BCR)DOWNSTREAM_SIGNALING_EVENTS_OF_B_CELL_RECEPTOR_BCR |
|  | TCR downstream signaling | 101 | 93 | [REACTOME_DOWNSTREAM_TCR_SIGNALING](http://www.gsea-msigdb.org/gsea/msigdb/cards/REACTOME_DOWNSTREAM_TCR_SIGNALING) |
| Programmed cell death | Programmed cell death | 178 | 175 | [REACTOME_PROGRAMMED_CELL_DEATH](http://www.gsea-msigdb.org/gsea/msigdb/cards/REACTOME_PROGRAMMED_CELL_DEATH) |
|  | PD-1 signaling | 27 | 20 | REACTOME_PD_1_SIGNALING |
| Interferon pathway | Interferon signaling | 202 | 184 | REACTOME_INTERFERON_SIGNALING |
|  | Interferon alpha response | 97 | 92 | HALLMARK_INTERFERON_ALPHA_RESPONSE |
|  | Interferon gamma response | 200 | 196 | HALLMARK_INTERFERON_GAMMA_RESPONSE |
| TGF-beta pathway | TGF-beta signaling | 54 | 54 | HALLMARK_TGF_BETA_SIGNALING |
| NOTCH pathway | NOTCH signaling | 32 | 32 | HALLMARK_NOTCH_SIGNALING |
|  | NOTCH1 signaling | 73 | 72 | REACTOME_SIGNALING_BY_NOTCH1 |
|  | NOTCH1 signaling in cancer | 58 | 57 | REACTOME_SIGNALING_BY_NOTCH1_IN_CANCER |
|  | NOTCH1: transmitting signal to the nucleus | 31 | 30 | REACTOME_ACTIVATED_NOTCH1_TRANSMITS_SIGNAL_TO_THE_NUCLEUS |
|  | NOTCH1: intracellular domain regulates transcription | 47 | 47 | REACTOME_NOTCH1_INTRACELLULAR_DOMAIN_REGULATES_TRANSCRIPTION |
|  | NOTCH2 signaling | 33 | 32 | REACTOME_SIGNALING_BY_NOTCH2 |
|  | NOTCH2: transmitting signal to the nucleus | 22 | 21 | REACTOME_NOTCH2_ACTIVATION_AND_TRANSMISSION_OF_SIGNAL_TO_THE_NUCLEUS |
|  | ~~NOTCH2: intracellular domain regulates transcription~~ | ~~12~~ | ~~Rejected~~ | ~~REACTOME_NOTCH2_INTRACELLULAR_DOMAIN_REGULATES_TRANSCRIPTION~~ |
|  | NOTCH3 signaling | 49 | 48 | REACTOME_SIGNALING_BY_NOTCH3 |
|  | NOTCH3: transmitting signal to the nucleus | 25 | 24 | REACTOME_NOTCH3_ACTIVATION_AND_TRANSMISSION_OF_SIGNAL_TO_THE_NUCLEUS |
|  | NOTCH3: intracellular domain regulates transcription | 25 | 25 | REACTOME_NOTCH3_INTRACELLULAR_DOMAIN_REGULATES_TRANSCRIPTION |
|  | NOTCH4 signaling | 82 | 81 | REACTOME_SIGNALING_BY_NOTCH4 |
|  | ~~NOTCH4: transmitting signal to the nucleus~~ | ~~11~~ | ~~Rejected~~ | ~~REACTOME_NOTCH4_ACTIVATION_AND_TRANSMISSION_OF_SIGNAL_TO_THE_NUCLEUS~~ |
|  | NOTCH4: intracellular domain regulates transcription | 20 | 20 | REACTOME_NOTCH4_INTRACELLULAR_DOMAIN_REGULATES_TRANSCRIPTION |
|  | Negative regelation of NOTCH4 signaling | 54 | 53 | REACTOME_NEGATIVE_REGULATION_OF_NOTCH4_SIGNALING |
|  | NOTCH-HLH transcription pathway | 28 | 28 | REACTOME_NOTCH_HLH_TRANSCRIPTION_PATHWAY |
| Interleukin pathway | IL-1 | 102 | 101 | [REACTOME_INTERLEUKIN_1_SIGNALING](http://www.gsea-msigdb.org/gsea/msigdb/cards/REACTOME_INTERLEUKIN_1_SIGNALING) |
|  | IL-1 family | 139 | 131 | [REACTOME_INTERLEUKIN_1_FAMILY_SIGNALING](http://www.gsea-msigdb.org/gsea/msigdb/cards/REACTOME_INTERLEUKIN_1_FAMILY_SIGNALING) |
|  | ~~IL-2~~ | ~~12~~ | ~~Rejected~~ | ~~REACTOME_INTERLEUKIN_2_SIGNALING~~ |
|  | IL-2 family | 44 | 43 | REACTOME_INTERLEUKIN_2_FAMILY_SIGNALING |
|  | IL-3/5 and GM-CSF | 48 | 47 | REACTOME_INTERLEUKIN_3_INTERLEUKIN_5_AND_GM_CSF_SIGNALING |
|  | IL-4/13 | 111 | 107 | REACTOME_INTERLEUKIN_4_AND_INTERLEUKIN_13_SIGNALING |
|  | ~~IL-6~~ | ~~11~~ | ~~Rejected~~ | ~~REACTOME_INTERLEUKIN_6_SIGNALING~~ |
|  | IL-6 family | 24 | 24 | REACTOME_INTERLEUKIN_6_FAMILY_SIGNALING |
|  | IL-6-JAK-STAT3 | 87 | 86 | HALLMARK_IL6_JAK_STAT3_SIGNALING |
|  | IL-7 | 34 | 33 | [REACTOME_INTERLEUKIN_7_SIGNALING](http://www.gsea-msigdb.org/gsea/msigdb/cards/REACTOME_INTERLEUKIN_7_SIGNALING) |
|  | ~~IL-9~~ | ~~9~~ | ~~Rejected~~ | ~~REACTOME_INTERLEUKIN_9_SIGNALING~~ |
|  | IL-10 | 46 | 45 | REACTOME_INTERLEUKIN_10_SIGNALING |
|  | IL-12 | 47 | 45 | [REACTOME_INTERLEUKIN_12_SIGNALING](http://www.gsea-msigdb.org/gsea/msigdb/cards/REACTOME_INTERLEUKIN_12_SIGNALING) |
|  | IL-12-JAK-STAT |  | 36 | REACTOME_GENE_AND_PROTEIN_EXPRESSION_BY_JAK_STAT_SIGNALING_AFTER_INTERLEUKIN_12_STIMULATION |
|  | IL-12 family | 57 | 55 | [REACTOME_INTERLEUKIN_12_FAMILY_SIGNALING](http://www.gsea-msigdb.org/gsea/msigdb/cards/REACTOME_INTERLEUKIN_12_FAMILY_SIGNALING) |
|  | ~~IL-15~~ | ~~14~~ | ~~Rejected~~ | ~~REACTOME_INTERLEUKIN_15_SIGNALING~~ |
|  | IL-17 | 71 | 70 | [REACTOME_INTERLEUKIN_17_SIGNALING](http://software.broadinstitute.org/gsea/msigdb/cards/REACTOME_INTERLEUKIN_17_SIGNALING.html) |
|  | ~~IL-18~~ | ~~8~~ | ~~Rejected~~ | ~~REACTOME_INTERLEUKIN_18_SIGNALING~~ |
|  | IL-20 family | 26 | 22 | [REACTOME_INTERLEUKIN_20_FAMILY_SIGNALING](http://software.broadinstitute.org/gsea/msigdb/cards/REACTOME_INTERLEUKIN_20_FAMILY_SIGNALING.html) |
|  | ~~IL-21~~ | ~~10~~ | ~~Rejected~~ | ~~REACTOME_INTERLEUKIN_21_SIGNALING~~ |
|  | ~~IL-23~~ | ~~9~~ | ~~Rejected~~ | ~~REACTOME_INTERLEUKIN_23_SIGNALING~~ |
|  | ~~IL-27~~ | ~~11~~ | ~~Rejected~~ | ~~REACTOME_INTERLEUKIN_27_SIGNALING~~ |
|  | ~~IL-35~~ | ~~12~~ | ~~Rejected~~ | ~~REACTOME_INTERLEUKIN_35_SIGNALLING~~ |
|  | ~~IL-36~~ | ~~6~~ | ~~Rejected~~ | ~~REACTOME_INTERLEUKIN_36_PATHWAY~~ |
|  | IL-37 | 21 | 19 | [REACTOME_INTERLEUKIN_37_SIGNALING](http://software.broadinstitute.org/gsea/msigdb/cards/REACTOME_INTERLEUKIN_37_SIGNALING.html) |
|  | Others | 288 | 279 | [REACTOME_OTHER_INTERLEUKIN_SIGNALING](http://www.gsea-msigdb.org/gsea/msigdb/cards/REACTOME_OTHER_INTERLEUKIN_SIGNALING) |

The lists consisting of less than 15 genes were not analyzed, due to the insufficient power of statistical test (highlighted in red).

## Supplementary Table S5. Clinicopathological and genomic characteristics in the surgery cohort.

|  |  | **Concordant dMMR/MSI-H**  **(n=115)** | **Concordant pMMR/MSS**  **(n=46)** | **Discordant (n=14)** | ***P* value**  **(dMMR/MSI-H vs. pMMR/MSS)** |
| --- | --- | --- | --- | --- | --- |
| ***Clinicopathological features*** | |  |  |  |  |
| Age |  |  |  |  | 0.22 |
|  | >65 | 54 (47.0%) | 16 (34.8%) | 8 (57.1%) |  |
|  | ≤65 | 61 (53.0%) | 30 (65.2%) | 6 (42.9%) |  |
| Sex |  |  |  |  | 0.15 |
|  | Male | 70 (60.9%) | 34 (73.9%) | 8 (57.1%) |  |
|  | Female | 45 (39.1%) | 12 (26.1%) | 6 (42.9%) |  |
| TNM stage |  |  |  |  | 0.050 |
|  | I | 29 (25.2%) | 7 (15.2%) | 4 (28.6%) |  |
|  | II | 48 (41.7%) | 13 (28.3%) | 5 (35.7%) |  |
|  | III | 37 (22.2%) | 25 (54.3%) | 5 (35.7%) |  |
|  | IV | 1 (0.9%) | 1 (2.2%) | 0 (0.0%) |  |
| Differentiation |  |  |  |  | 0.96 |
|  | Poor/undifferentiated | 80 (69.5%) | 29 (63.0%) | 8 (57.2%) |  |
|  | Moderate | 30 (26.0%) | 14 (30.5%) | 5 (35.7%) |  |
|  | Well | 2 (1.7%) | 1 (2.2%) | 1 (7.1%) |  |
|  | N.A. | 3 (2.6%) | 2 (4.3%) | 0 (0.0%) |  |
| Lauren |  |  |  |  | 0.058 |
|  | Intestinal | 59 (51.3%) | 15 (32.6%) | 7 (50.0%) |  |
|  | Diffuse | 19 (16.5%) | 14 (30.4%) | 1 (7.1%) |  |
|  | Mixed | 36 (31.3%) | 16 (34.8%) | 6 (42.9%) |  |
|  | N.A. | 1 (0.9%) | 1 (2.2%) | 0 (0.0%) |  |
| HER2 (IHC) |  |  |  |  | 0.034 |
|  | 0 | 51 (44.3%) | 19 (41.3%) | 6 (42.9%) |  |
|  | 1+ | 32 (27.8%) | 10 (21.7%) | 5 (35.7%) |  |
|  | 2+* | 30 (26.1%) | 7 (15.2%) | 3 (21.4%) |  |
|  | 3+ | 2 (1.7%) | 5 (10.9%) | 0 (0.0%) |  |
|  | N.A. | 0 (0.0%) | 5 (10.9%) | 0 (0.0%) |  |
| EBV (FISH) |  |  |  |  | 0.008 |
|  | Positive | 1 (0.9%) | 5 (10.9%) | 0 (0.0%) |  |
|  | Negative | 113 (98.3%) | 41 (89.1%) | 14 (100.0%) |  |
|  | N.A. | 1 (0.9%) | 0 (0.0%) | 0 (0.0%) |  |
| ***IHC testing of MMR protein*** | |  |  |  |  |
| MMR (IHC) |  |  |  |  | 2.1*10^-41^ |
|  | dMMR | 115 (100.0%) | 0 (0.0%) | 10 (71.4%) |  |
|  | Indeterminate | 0 (0.0%) | 0 (0.0%) | 4 (28.6%) |  |
|  | pMMR | 0 (0.0%) | 46 (100.0%) | 0 (0.0%) |  |
| MLH1 |  |  |  |  | 4.9*10^-36^ |
|  | Complete loss | 111 (96.5%) | 0 (0.0%) | 9 (64.3%) |  |
|  | Incomplete loss | 0 (0.0%) | 0 (0.0%) | 4 (28.6%) |  |
|  | No loss | 4 (3.5%) | 46 (100%) | 1 (7.1%) |  |
| PMS2 |  |  |  |  | 7.6*10^-31^ |
|  | Complete loss | 105 (91.3%) | 0 (0.0%) | 9 (64.3%) |  |
|  | Incomplete loss | 0 (0.0%) | 0 (0.0%) | 4 (28.6%) |  |
|  | No loss | 10 (8.7%) | 46 (100%) | 1 (7.1%) |  |
| MSH2 |  |  |  |  | 0.68 |
|  | Complete loss | 3 (2.6%) | 0 (0.0%) | 0 (0.0%) |  |
|  | Incomplete loss | 1 (0.9%) | 0 (0.0%) | 0 (0.0%) |  |
|  | No loss | 111 (96.5%) | 46 (100%) | 14 (100%) |  |
| MSH6 |  |  |  |  | 0.33 |
|  | Complete loss | 3 (2.6%) | 0 (0.0%) | 0 (0.0%) |  |
|  | Incomplete loss | 3 (2.6%) | 0 (0.0%) | 1 (7.1%) |  |
|  | No loss | 109 (94.8%) | 46 (100%) | 13 (92.9%) |  |
| ***PCR testing of 5 MS loci*** | |  |  |  |  |
| MSI-H (PCR) |  |  |  |  | 2.1*10^-41^ |
|  | MSI-H | 115 (100.0%) | 0 (0.0%) | 1 (7.1%) |  |
|  | MSI-L | 0 (0.0%) | 0 (0.0%) | 5 (35.7%) |  |
|  | MSS | 0 (0.0%) | 46 (100.0%) | 8 (57.1%) |  |
| BAT26 | Unstable | 111 (96.5%) | 0 (0.0%) | 3 (21.4%) | 3.4*10^-39^ |
| NR24 | Unstable | 86 (74.8%) | 0 (0.0%) | 1 (7.1%) | 1.6*10^-23^ |
| NR21 | Unstable | 102 (88.7%) | 0 (0.0%) | 0 (14.3%) | 3.8*10^-32^ |
| MONO27 | Unstable | 104 (90.4%) | 0 (0.0%) | 3 (21.4%) | 1.1*10^-32^ |
| BAT25 | Unstable | 90 (78.3%) | 0 (0.0%) | 1 (7.1%) | 7.2*10^-26^ |
| ***NGS testing of 100 MS loci*** | |  |  |  |  |
| MSI-H (NGS) |  |  |  |  | 2.1*10^-41^ |
|  | MSI-H | 115 (100.0%) | 0 (0.0%) | 7 (50.0%) |  |
|  | MSS | 0 (0.0%) | 46 (100.0%) | 7 (50.0%) |  |
| MSI score |  | 97 (91-99) | 2.0 (1.0-5.0) | 36 (5.0-64) | 2.1*10^-41^ |
| ***NGS testing of 733-gene panel*** | |  |  |  |  |
| TMB |  | 65 (49-90) | 6.1 (3.4-9.2) | 17 (4.9-33) | 7.2*10^-23^ |
| Frameshift |  | 36 (25-44) | 0 (0-1) | 6.5 (1.5-14.75) | 3.6*10^-23^ |
| Missense |  | 49 (37-69) | 6.5 (3-9) | 14.5 (5.75-28.75) | 1.7*10^-22^ |
| Non-frameshift indel |  | 2 (1-4) | 0 (0-1) | 0 (0-1.25) | 4.2*10^-13^ |
| Stopgain |  | 4 (2-7) | 1 (0-2) | 0.5 (0-2) | 2.3*10^-15^ |
| Stoploss |  | 0 (0-0) | 0 (0-0) | 0 (0-0) | 0.20 |
| Splice site |  | 1 (1-3) | 0 (0-1) | 1 (0-1) | 3.0*10^-10^ |
| TSS loss |  | 0 (0-0) | 0 (0-0) | 0 (0-0) | 0.20 |
| Gain |  | 0 (0-0) | 0 (0-2) | 0 (0-1) | 3.6*10^-10^ |
| Loss |  | 0 (0-0) | 0 (0-0) | 0 (0-0) | 0.21 |
| Fusion |  | 0 (0-0) | 0 (0-1) | 0 (0-1) | 0.094 |

*All IHC-HER2 2+ samples showed no *ERBB2* amplification in the NGS testing.

Data are n (%) or median (IQR). Abbreviations: dMMR=mismatch repair-deficient, EBV=Epstein-Barr virus, FISH=fluorescence *in situ* hybridization, HER2=human epidermal growth factor receptor 2, IHC=immunohistochemistry, MLH1=MutL homolog 1, MMR=mismatch repair, MSH2=MutS Homolog 2, MSH6=MutS Homolog 6, MSI-H=microsatellite instability-high, MSI-L=microsatellite instability-low, MSS=microsatellite stability, N.A.=not applicable, NGS=next-generation sequencing, PCR=polymerase chain reaction, PMS2=PMS1 Homolog 2, TMB=tumor mutational burden, TSS=transcription start site.

## Supplementary Table S6. Association between the results of IHC, PCR, and NGS testing for identifying dMMR/MSI-H gastric adenocarcinoma.

|  |  |  | Consistency between  PCR and IHC | **NGS (100-loci MSI)** | |
| --- | --- | --- | --- | --- | --- |
|  |  |  |  | NGS-MSS | NGS-MSI-H |
| **PCR (5-loci)**  **& IHC** | PCR-MSS | IHC-pMMR | **Concordant** | 46 | 0 |
|  |  | IHC-heterogeneity | Discordant | 3 | 0 |
|  |  | IHC-dMMR | Discordant | 5 | 0 |
|  | PCR-MSI-L | IHC-pMMR | Discordant | 0 | 0 |
|  |  | IHC-heterogeneity | Discordant | 0 | 0 |
|  |  | IHC-dMMR | Discordant | 1 | 4 |
|  | PCR-MSI-H | IHC-pMMR | Discordant | 0 | 0 |
|  |  | IHC-heterogeneity | Discordant | 0 | 1 |
|  |  | IHC-dMMR | **Concordant** | 0 | 115 |

Abbreviations: dMMR=mismatch repair-deficient, IHC=immunohistochemistry, MSI-H=microsatellite instability-high, MSI-L=microsatellite instability-low, MSS=microsatellite stability, NGS=next-generation sequencing, PCR=polymerase chain reaction, pMMR=mismatch repair-proficient.

## Supplementary Table S7 Genomic characteristics of the discordant samples.

| **No.** | **IHC staining (MLH1, PMS2, MSH2, MSH6)** | **IHC result** | **Tumor purity %** | **PCR: no. of**  **unstable loci** | **PCR result**  **(0:MSS; 1: MSI-L; ≥2: MSI-H)** | **NGS:**  **MSI score** | **NGS result**  **(≥30: MSI-H)** | **TMB** | **Frameshift count** |
| --- | --- | --- | --- | --- | --- | --- | --- | --- | --- |
| **9** | **MLH1 heterogeneity + PMS2 heterogeneity** | **Heterogeneity** | **25** | **5** | **MSI-H** | **87** | **MSI-H** | **56.98** | **30** |
| 27 | MLH1 loss + PMS2 loss | dMMR | 40 | 1 | MSI-L | 24 | MSS | 8.38 | 3 |
| 35 | MLH1 loss + PMS2 loss | dMMR | 20 | 0 | MSS | 25 | MSS | 5.03 | 2 |
| **36** | **MLH1 loss + PMS2 loss** | **dMMR** | **50** | **0** | **MSS** | **52** | **MSI-H** | **25.14** | **8** |
| **58** | **MLH1 loss + PMS2 loss** | **dMMR** | **30** | **1** | **MSI-L** | **88** | **MSI-H** | **61.45** | **34** |
| 63 | MLH1 heterogeneity + PMS2 heterogeneity | Heterogeneity | 60 | 0 | MSS | 0 | MSS | 8.38 | 2 |
| **72** | **MLH1 loss + PMS2 loss** | **dMMR** | **20** | **1** | **MSI-L** | **46** | **MSI-H** | **28.49** | **20** |
| 78 | MLH1 loss + PMS2 loss | dMMR | 20 | 0 | MSS | 5 | MSS | 0.56 | 0 |
| **90** | **MLH1 loss + PMS2 loss** | **dMMR** | **40** | **1** | **MSI-L** | **68** | **MSI-H** | **17.32** | **7** |
| **127** | **MLH1 loss + PMS2 loss** | **dMMR** | **70** | **1** | **MSI-L** | **63** | **MSI-H** | **28.49** | **13** |
| 129 | PMS2 heterogeneity | Heterogeneity | 75 | 0 | MSS | 1 | MSS | 4.47 | 0 |
| 130 | MLH1 heterogeneity + PMS2 heterogeneity | Heterogeneity | 30 | 0 | MSS | 5 | MSS | 3.35 | 0 |
| 132 | MSH6 heterogeneity | Heterogeneity | 60 | 0 | MSS | 21 | MSS | 46.37* | 6 |
| **145** | **MLH1 loss + PMS2 heterogeneity** | **dMMR** | **40** | **0** | **MSS** | **58** | **MSI-H** | **16.20** | **7** |

*The sample of patient #132 harbored a *POLE* mutation and a *MSH6* mutation and exhibited incomplete loss of MSH6 and PCR/NGS-MSS. The *POLE* mutation might be on account of the high single nucleotide variation burden.

The discordant samples identified as MSI-H by NGS are highlighted in bold. Abbreviations: IHC=immunohistochemistry, MLH1=MutL homolog 1, MSH2=MutS Homolog 2, MSH6=MutS Homolog 6, MSI-L=microsatellite instability-low, MSI-H=microsatellite instability-high, MSS=microsatellite stability, NGS=next-generation sequencing, PCR=polymerase chain reaction, PMS2=PMS1 Homolog 2, TMB=tumor mutational burden.

## Supplementary Table S8. Clinicopathological characteristics of concordant dMMR/MSI-H samples according to the evaluation of immune infiltration in the surgery cohort.

|  |  | **Total**  **(n=115)** | **With data of immune infiltration (n=103)** | **Without data of immune infiltration (n=12)** | ***P* value** |
| --- | --- | --- | --- | --- | --- |
| Age |  |  |  |  | 0.37 |
|  | >65 | 54 (47.0%) | 50 (48.5%) | 8 (66.7%) |  |
|  | ≤65 | 61 (53.0%) | 53 (51.5%) | 4 (33.3%) |  |
| Sex |  |  |  |  | 0.36 |
|  | Male | 70 (60.9%) | 61 (59.2%) | 9 (75.0%) |  |
|  | Female | 45 (39.1%) | 42 (40.8%) | 3 (25.0%) |  |
| TNM stage |  |  |  |  | 0.51 |
|  | I | 29 (25.2%) | 26 (25.2%) | 3 (25.0%) |  |
|  | II | 48 (41.7%) | 45 (43.7%) | 3 (25.0%) |  |
|  | III | 37 (22.2%) | 31 (30.1%) | 6 (50.0%) |  |
|  | IV | 1 (0.9%) | 1 (1.0%) | 0 (0.0%) |  |
| Differentiation |  |  |  |  | 0.78 |
|  | Poor | 80 (69.5%) | 72 (69.9%) | 8 (66.7%) |  |
|  | Moderate | 30 (26.0%) | 28 (27.4%) | 2 (16.7%) |  |
|  | Well | 2 (1.7%) | 2 (1.9%) | 0 (0.0%) |  |
|  | N.A. | 3 (2.6%) | 1 (1.0%) | 2 (16.7%) |  |
| Lauren |  |  |  |  | 0.65 |
|  | Intestinal | 59 (51.3%) | 55 (53.4%) | 4 (33.3%) |  |
|  | Diffuse | 19 (16.5%) | 19 (16.5%) | 2 (16.7%) |  |
|  | Mixed | 36 (31.3%) | 36 (31.3%) | 5 (41.7%) |  |
|  | N.A. | 1 (0.9%) | 0 (0.0%) | 1 (8.3%) |  |
| HER2 (IHC) |  |  |  |  | 0.70 |
|  | 0 | 51 (44.3%) | 44 (42.7%) | 7 (58.3%) |  |
|  | 1+ | 32 (27.8%) | 30 (29.1%) | 2 (16.7%) |  |
|  | 2+* | 30 (26.1%) | 27 (26.2%) | 3 (25.0%) |  |
|  | 3+ | 2 (1.7%) | 2 (1.9%) | 0 (0.0%) |  |
|  | N.A. | 0 (0.0%) | 0 (0.0%) | 0 (0.0%) |  |
| EBV (FISH) |  |  |  |  | >0.99 |
|  | Positive | 1 (0.9%) | 1 (1.0%) | 0 (0.0%) |  |
|  | Negative | 113 (98.3%) | 102 (99.0%) | 11 (91.7%) |  |
|  | N.A. | 1 (0.9%) | 0 (0.0%) | 1 (8.3%) |  |

*All IHC-HER2 2+ samples showed no *ERBB2* amplification in the NGS testing.

Data are n (%). Abbreviations: dMMR=mismatch repair-deficient, EBV=Epstein-Barr virus, FISH=fluorescence *in situ* hybridization, HER2=human epidermal growth factor receptor 2, IHC=immunohistochemistry, MSI-H=microsatellite instability-high, N.A.=not applicable.

## Supplementary Table S9. Clinicopathological characteristics of dMMR/MSI-H samples with evaluation of immune infiltration according to the history of neoadjuvant chemotherapy in the surgery cohort.

|  |  | **Total**  **(n=103)** | **Not received** **neoadjuvant chemotherapy (n=89)** | **Received** **neoadjuvant chemotherapy (n=14)** | ***P* value** |
| --- | --- | --- | --- | --- | --- |
| Age |  |  |  |  | 0.39 |
|  | >65 | 50 (48.5%) | 45 (50.6%) | 5 (35.7%) |  |
|  | ≤65 | 53 (51.5%) | 44 (49.4%) | 9 (64.3%) |  |
| Sex |  |  |  |  | 0.39 |
|  | Male | 61 (59.2%) | 51 (57.3%) | 4 (28.6%) |  |
|  | Female | 42 (40.8%) | 38 (42.7%) | 10 (71.4%) |  |
| TNM stage |  |  |  |  | 0.54 |
|  | I | 26 (25.2%) | 24 (27.0%) | 2 (14.3%) |  |
|  | II | 45 (43.7%) | 39 (43.8%) | 6 (42.9%) |  |
|  | III | 31 (30.1%) | 25 (28.1%) | 6 (42.9%) |  |
|  | IV | 1 (1.0%) | 1 (1.1%) | 0 (0.0%) |  |
| Differentiation |  |  |  |  | 0.003 |
|  | Poor | 72 (69.9%) | 59 (66.3%) | 13 (92.9%) |  |
|  | Moderate | 28 (28.1%) | 28 (31.4%) | 0 (0.0%) |  |
|  | Well | 2 (1.9%) | 2 (2.2%) | 0 (0.0%) |  |
|  | N.A. | 1 (1.0%) | 0 (0.0%) | 1 (7.1%) |  |
| Lauren |  |  |  |  | 0.14 |
|  | Intestinal | 55 (53.4%) | 49 (55.1%) | 6 (42.9%) |  |
|  | Diffuse | 19 (16.5%) | 12 (13.5%) | 5 (35.7%) |  |
|  | Mixed | 36 (31.3%) | 28 (28.3%) | 3 (21.4%) |  |
|  | N.A. | 0 (0.0%) | 0 (0.0%) | 0 (0.0%) |  |
| HER2 (IHC) |  |  |  |  | 0.37 |
|  | 0 | 44 (42.7%) | 35 (39.3%) | 9 (64.3%) |  |
|  | 1+ | 30 (29.1%) | 28 (31.5%) | 2 (14.3%) |  |
|  | 2+* | 27 (26.2%) | 24 (27.0%) | 3 (21.4%) |  |
|  | 3+ | 2 (1.9%) | 2 (2.2%) | 0 (0.0%) |  |
|  | N.A. | 0 (0.0%) | 0 (0.0%) | 0 (0.0%) |  |
| EBV (FISH) |  |  |  |  | 1.00 |
|  | Positive | 1 (1.0%) | 1 (1.1%) | 0 (0.0%) |  |
|  | Negative | 102 (99.0%) | 88 (98.9%) | 14 (100.0%) |  |
|  | N.A. | 0 (0.0%) | 0 (0.0%) | 0 (0.0%) |  |

*All IHC-HER2 2+ samples showed no *ERBB2* amplification in the NGS testing.

Data are n (%). Abbreviations: dMMR=mismatch repair-deficient, EBV=Epstein-Barr virus, FISH=fluorescence *in situ* hybridization, HER2=human epidermal growth factor receptor 2, IHC=immunohistochemistry, MSI-H=microsatellite instability-high, N.A.=not applicable.

## Supplementary Table S10 Sensitivity analysis of the correlation between mutations in PI3K-AKT-mTOR pathway and immune cell infiltration.

| Omitted group (number of mutated members in PI3K-AKT-mTOR pathway) |  | CD3  central tumor | CD3  invasive margin | CD4  central tumor | CD4  invasive margin | CD8  central tumor | CD8  invasive margin | CD68  central tumor | CD68  invasive margin | FOXP3  central tumor | FOXP3  invasive margin |
| --- | --- | --- | --- | --- | --- | --- | --- | --- | --- | --- | --- |
| 0 | Spearman rho | -0.3006 | -0.2470 | -0.1516 | -0.0270 | -0.2903 | -0.1247 | -0.0441 | 0.0619 | -0.2369 | -0.0961 |
|  | *p* value | **0.0071** | **0.0282** | 0.1822 | 0.8135 | **0.0095** | 0.2737 | 0.6999 | 0.5878 | **0.0355** | 0.3993 |
| 1 | Spearman rho | -0.278 | -0.1373 | -0.0432 | 0.0631 | -0.2233 | -0.0844 | -0.0108 | 0.1762 | -0.1294 | -0.0179 |
|  | *p* value | **0.0238** | 0.2717 | 0.7308 | 0.6146 | **0.0715** | 0.5004 | 0.9311 | 0.1570 | 0.3003 | 0.8865 |
| 2 | Spearman rho | -0.3948 | -0.2426 | -0.1895 | -0.0475 | -0.3595 | -0.1114 | -0.1066 | 0.1424 | -0.2598 | -0.1007 |
|  | *p* value | **0.0011** | **0.0516** | 0.1307 | 0.7072 | **0.0033** | 0.3770 | 0.3982 | 0.2578 | **0.0366** | 0.4248 |
| 3 | Spearman rho | -0.3091 | -0.1904 | -0.204 | -0.0723 | -0.3158 | -0.0923 | -0.1281 | 0.1202 | -0.2495 | -0.1062 |
|  | *p* value | **0.0074** | 0.1041 | **0.0813** | 0.5404 | **0.0061** | 0.4340 | 0.2769 | 0.3078 | **0.0320** | 0.3680 |
| 4 | Spearman rho | -0.3899 | -0.2364 | -0.2735 | -0.1100 | -0.2902 | -0.0452 | -0.2293 | 0.1021 | -0.2199 | -0.0774 |
|  | *p* value | **0.0004** | **0.0359** | **0.0147** | 0.3347 | **0.0095** | 0.6927 | **0.0421** | 0.3704 | **0.0515** | 0.4980 |
| 5 | Spearman rho | -0.3337 | -0.2209 | -0.1850 | -0.0516 | -0.3027 | -0.1339 | -0.0987 | 0.1256 | -0.2295 | -0.1189 |
|  | *p* value | **0.0019** | **0.0434** | **0.0921** | 0.6410 | **0.0051** | 0.2245 | 0.3718 | 0.2549 | **0.0358** | 0.2812 |
| 6 | Spearman rho | -0.3515 | -0.2199 | -0.2354 | -0.0793 | -0.2921 | -0.1079 | -0.0965 | 0.1555 | -0.2146 | -0.0835 |
|  | *p* value | **0.0008** | **0.0396** | **0.0273** | 0.4628 | **0.0058** | 0.3171 | 0.3709 | 0.1479 | **0.0446** | 0.4390 |

*P* values below 0.10 are highlighted in bold.

## Supplementary Table S11. Detailed information of the MSI-H gastric adenocarcinoma samples evaluated by both 381-gene panel and PD-L1 kit retrieved from the 3DMed database.

| **No.** | **Age** | **Sex** | **TMB** | **TPS** | **IPS** | **CPS** | **Sum of mutations**  **in PI3K-AKT-mTOR pathway** |
| --- | --- | --- | --- | --- | --- | --- | --- |
| CP60000283 | 63 | Female | 65.8887 | 0 | 0 | 0 | 3 |
| CP60003089 | 58 | Male | 69.8718 | 0 | 0 | 0 | 1 |
| CP60007085 | 50 | Female | 9.67742 | 0 | 0 | 0 | 0 |
| CP60008445 | 67 | Male | 75 | 10 | 0 | 10 | 3 |
| CP60010313 | 31 | Female | 51.6129 | 1 | 0 | 1 | 3 |
| CP60012392 | 84 | Female | 87.9032 | 5 | 0 | 5 | 3 |
| CP60016394 | 82 | Female | 69.3548 | 0 | 0 | 0 | 2 |
| **CP60018286** | **52** | **Female** | **63.7097** | **50** | **0** | **50** | **4** |
| CP60021616 | 63 | Male | 100 | 0 | 0 | 0 | 3 |
| CP60022246 | 39 | Female | 28.2258 | 0 | 0 | 0 | 0 |
| CP60023908 | 66 | Male | 46.7742 | 1 | 0 | 1 | 1 |
| CP60023938 | 62 | Female | 20.1613 | 0 | 0 | 0 | 1 |
| CP70001324 | 66 | Female | 86.2903 | 1 | 0 | 1 | 4 |
| **CP70008370** | **70** | **Male** | **58.871** | **0** | **0** | **0** | **2** |
| CP70009941 | 78 | Male | 87.9032 | 0 | 0 | 0 | 2 |
| CP70010610 | 56 | Male | 37.0968 | 40 | 10 | 42 | 0 |
| CP70011643 | 50 | Male | 25 | 5 | 25 | 10 | 0 |
| CP70012903 | 81 | Male | 49.162 | 0 | 0 | 0 | 1 |
| CP70013233 | 39 | Male | 104.839 | 0 | 0 | 0 | 3 |
| CP70015125 | 39 | Female | 36.2903 | 10 | 0 | 10 | 2 |
| **CP70016821** | **83** | **Female** | **73.3871** | **55** | **15** | **60** | **1** |
| CP70019541 | 68 | Male | 58.1006 | 0 | 10 | 3 | 3 |
| CP70022015 | 62 | Female | 83.871 | 0 | 0 | 0 | 1 |
| CP70023317 | 58 | Male | 54.8387 | 0 | 20 | 5 | 2 |
| CP70024678 | 71 | Male | 68.5484 | 5 | 15 | 10 | 3 |
| CP70024702 | 42 | Female | 26.6129 | 10 | 25 | 15 | 1 |
| CP70024951 | 58 | Male | 66.9355 | 0 | 2 | 5 | 5 |
| CP70025367 | 72 | Female | 51.3966 | 10 | 35 | 17 | 0 |
| CP70028458 | 57 | Male | 169.355 | 1 | 15 | 5 | 6 |
| CP70038412 | 65 | Female | 109.497 | 10 | 5 | 11 | 3 |
| CP70038915 | 61 | Female | 42.4581 | 0 | 0 | 0 | 1 |
| CP70047861 | 67 | Male | 48.0447 | 0 | 0 | 0 | 3 |

The texts of the samples whose images were shown in the Fig. 4C are highlighted in bold. Abbreviations: CPS=combined positive score, IPS=immune proportion score, PD-L1=programmed death-ligand 1, TPS=tumor proportion score.

## Supplementary Table S12. Clinicopathological characteristics of MSI-H STAD according to the genetic aberration in PI3K-AKT-mTOR pathway in the TCGA cohort

|  |  | **Total**  **(n=73)** | **PI3K-AKT-mTOR^WT^**  **(n=21)** | **PI3K-AKT-mTOR^mut^**  **(n=52)** | ***P* value** |
| --- | --- | --- | --- | --- | --- |
| Age |  |  |  |  | 0.39 |
|  | >65 | 51 (69.9%) | 13 (61.9%) | 38 (73.1%) |  |
|  | ≤65 | 21 (28.8%) | 8 (38.1%) | 13 (25.0%) |  |
|  | N.A. | 1 (1.4%) | 0 (0.0%) | 1 (1.9%) |  |
| Sex |  |  |  |  | 1.00 |
|  | Male | 35 (47.9%) | 10 (47.6%) | 25 (48.1%) |  |
|  | Female | 38 (52.1%) | 11 (52.4%) | 27 (51.9%) |  |
| Race |  |  |  |  | 0.50 |
|  | White | 44 (60.3%) | 10 (47.6%) | 34 (65.4%) |  |
|  | Asian | 15 (20.5%) | 6 (28.6%) | 9 (17.3%) |  |
|  | African | 1 (1.4%) | 0 (0.0%) | 1 (1.9%) |  |
|  | N.A. | 13 (17.8%) | 5 (23.8%) | 8 (15.4%) |  |
| TNM stage |  |  |  |  | 0.88 |
|  | I | 13 (17.8%) | 4 (19.0%) | 9 (17.3%) |  |
|  | II | 24 (32.9%) | 6 (28.6%) | 18 (34.6%) |  |
|  | III | 23 (31.5%) | 8 (38.1%) | 15 (28.8%) |  |
|  | IV | 6 (8.2%) | 2 (9.5%) | 4 (7.7%) |  |
|  | N.A. | 7 (9.6%) | 1 (4.8%) | 6 (11.5%) |  |
| Grade |  |  |  |  | 0.41 |
|  | G1 | 3 (4.1%) | 1 (4.8%) | 2 (3.8%) |  |
|  | G2 | 22 (30.1%) | 4 (19.0%) | 18 (34.6%) |  |
|  | G3 | 47 (64.4%) | 16 (76.2%) | 31 (59.6%) |  |
|  | N.A. | 1 (1.4%) | 0 (0.0%) | 1 (1.9%) |  |
| Residual tumor |  |  |  |  | 0.018 |
|  | R0 | 53 (72.6%) | 12 (57.1%) | 41 (78.8%) |  |
|  | R1 | 3 (4.1%) | 2 (9.5%) | 1 (1.9%) |  |
|  | R2 | 2 (2.7%) | 2 (9.5%) | 0 (0.0%) |  |
|  | RX | 10 (13.7%) | 2 (9.5%) | 8 (15.4%) |  |
|  | N.A. | 5 (6.8%) | 3 (14.3%) | 2 (3.8%) |  |

Data are n (%). Abbreviations: MSI-H=microsatellite instability-high, N.A.=not applicable, STAD=stomach adenocarcinoma.

## Supplementary Table S13. The mutations of the members in the PI3K-AKT-mTOR pathway and mutation sites in the five MSI-H GAC cell lines

| **Gene** | **NUGC-3** | **TGBC11TKB** | **SNU-1** | **IM95** | **23132/87** |
| --- | --- | --- | --- | --- | --- |
| PIK3CA |  | p.E545D |  | p.E542K |  |
| PIK3R1 |  |  |  |  |  |
| PIK3R2 |  |  |  | p.Q227R | p.N561D |
| PTEN |  |  |  |  |  |
| AKT1 |  |  |  |  | p.R406H |
| AKT2 |  |  | p.T372M |  |  |
| AKT3 |  |  |  | p.T298A | p.G265R |
| TSC1 |  |  |  | p.Y373C |  |
| TSC2 | p.V299Cfs*29 | p.A306Sfs*32 | |  |  |
| PDPK1 |  |  |  |  |  |
| MTOR |  |  | p.T431A |  | p.H262R |
| RICTOR |  |  | p.R1097H |  |  |
| RHEB |  |  |  |  |  |
| RPTOR | p.A964T |  | p.R788C |  |  |
| MLST8 |  |  |  |  | p.P52A |

Abbreviations: MSI-H=microsatellite instability-high, GAC=gastric adenocarcinomas.

## Supplementary Table S14. The IC50 values of every cell line for the PI3K-AKT-mTOR inhibitors, the inhibitors targeting VEGFR or EGFR, and chemotherapeutic drugs

|  | **Inhibitors** | **NUGC-3** | **TGBC11TKB** | **SNU-1** | **IM95** | **23132/87** |
| --- | --- | --- | --- | --- | --- | --- |
| PI3K-AKT-mTOR inhibitors | AKT inhibitor VIII 2/2 | 92.32 | 19.96 | 4.23 | 1.56 | 2.77 |
|  | GSK690693 | 60.17 | 29.16 | 75.33 | 3.30 | 9.50 |
|  | MK-2206 | 9.11 | 43.85 | 67.62 | NA | 0.56 |
|  | Temsirolimus | NA | 1.25 | 1.56 | NA | 0.06 |
|  | OSI-027 | 17.36 | 50.35 | 2.96 | 1.70 | 1.77 |
|  | AZD8055 | 0.53 | 1.70 | 10.35 | NA | 0.57 |
|  | OSU-03012 | 5.01 | 11.95 | 7.26 | 3.00 | 7.86 |
|  | BX-912 | 59.89 | 91.16 | 6.05 | 15.11 | 36.86 |
|  | KIN001-244 | 42.77 | 22.51 | 12.45 | 9.01 | 8.94 |
|  | Omipalisib | 0.12 | 3.19 | 0.06 | 0.01 | 0.02 |
|  | Dactolisib | 0.02 | 0.25 | 1.72 | NA | 0.15 |
|  | Pictilisib 2/2 | 2.69 | 9.95 | 14.67 | 4.29 | 0.33 |
|  | ZSTK474 | 6.26 | 31.31 | 1.50 | 0.54 | 1.06 |
|  | PI-103 | 4.37 | 99.81 | 0.59 | 15.61 | 1.04 |
|  | AZD6482 1/2 | 26.96 | 55.80 | 18.12 | 5.14 | 3.68 |
|  | Idelalisib | 166.96 | 138.41 | 69.34 | 32.28 | 83.98 |
|  | AS605240 | 49.08 | 76.49 | 4.74 | 2.07 | 19.43 |
|  | PIK-93 | 41.86 | 12.10 | 5.19 | 5.00 | 7.53 |
|  | PF-4708671 | 30.77 | 18.48 | 19.73 | 32.19 | 29.46 |
| VEGFR inhibitors | Ponatinib | 6.30 | 5.16 | 1.18 | 1.04 | 6.57 |
|  | Axitinib | NA | 6.29 | 21.60 | NA | 22.32 |
|  | Cabozantinib | 89.68 | 21.35 | 5.93 | 2.42 | 10.54 |
|  | Motesanib | 9.80 | 17.14 | 27.33 | NA | 6.02 |
|  | Linifanib | 10.47 | 7.50 | 9.15 | 7.68 | 15.38 |
|  | Tivozanib | 2.08 | 1.22 | 1.29 | 1.48 | 1.75 |
| Chemotherapeutic drugs | Doxorubicin | 0.05 | 2.07 | 0.07 | 0.05 | 0.02 |
|  | 5-Fluorouracil | 69.09 | 62.06 | 8.69 | 18.19 | 3.95 |
|  | Cisplatin | NA | 39.71 | 55.66 | NA | 59.86 |
|  | Mitomycin-C | 0.04 | 0.77 | 0.14 | 0.39 | 0.07 |
|  | Vinblastine | NA | 0.02 | 0.15 | NA | 0.01 |
|  | Vinorelbine | 0.01 | 0.03 | 0.03 | 0.00 | 0.01 |
|  | Epothilone B | 0.00 | 0.01 | 0.02 | 0.00 | 0.00 |
|  | Docetaxel | NA | 0.01 | 0.09 | NA | 0.01 |
|  | Gemcitabine | 0.02 | 0.14 | 0.00 | 0.02 | 0.05 |
|  | Etoposide | 1.15 | 42.95 | 1.90 | 4.90 | 0.61 |
| EGFR inhibitors | Pelitinib | 4.35 | 51.03 | 2.07 | 0.74 | 12.60 |
|  | Cetuximab | 46.80 | 359.50 | 874.25 | NA | 641.24 |
|  | Gefitinib | NA | 2.92 | 6.89 | NA | 7.54 |
|  | Afatinib 2/2 | 1.02 | 40.59 | 151.12 | NA | 75.77 |
|  | CUDC-101 | 0.52 | 2.21 | 0.31 | 0.43 | 0.24 |

Abbreviations: IC50=50% inhibitory concentration, NA=not applicable.

## Supplementary Table S15. Baseline characteristics of the patients with dMMR/MSI-H G/GEJ adenocarcinoma in the ICI treatment cohort.

| **Parameter** |  | **Total**  **(n=36)** | **NMP=0/1**  **(n=16)** | **NMP≥2**  **(n=20)** | ***P* value** |
| --- | --- | --- | --- | --- | --- |
| Age |  |  |  |  | 1.00 |
|  | >65 | 14 (38.9%) | 5 (31.3%) | 9 (45.0%) |  |
|  | ≤65 | 22 (61.1%) | 11 (68.8%) | 11 (55.0%) |  |
| Sex |  |  |  |  | 0.50 |
|  | Male | 14 (38.9%) | 6 (37.5%) | 8 (40.0%) |  |
|  | Female | 22 (61.1%) | 10 (62.5%) | 12 (60.0%) |  |
| Primary location |  |  |  |  | 0.61 |
|  | GEJ | 32 (88.9%) | 15 (93.8%) | 17 (85.0%) |  |
|  | Others | 4 (11.1%) | 1 (6.3%) | 3 (15.0%) |  |
| Pathology |  |  |  |  | 0.76 |
|  | Poor/undifferentiated | 27 (75.0%) | 10 (62.5%) | 17 (85.0%) |  |
|  | Moderate to well | 9 (25.0%) | 6 (37.5%) | 3 (15.0%) |  |
| Stage |  |  |  |  | 0.29 |
|  | Locally advanced | 10 (27.8%) | 6 (37.5%) | 4 (20.0%) |  |
|  | Metastatic | 26 (72.2%) | 10 (62.5%) | 16 (80.0%) |  |
| ECOG |  |  |  |  | 0.31 |
|  | 0 | 11 (30.6%) | 4 (25.0%) | 7 (35.0%) |  |
|  | 1 | 21 (58.3%) | 10 (62.5%) | 11 (55.0%) |  |
|  | 2 | 2 (5.6%) | 2 (12.5%) | 0 (0.0%) |  |
|  | 3 | 2 (5.6%) | 0 (0.0%) | 2 (10.0%) |  |
| Treatment lines |  |  |  |  | 0.12 |
|  | 1 | 17 (47.2%) | 5 (31.3%) | 12 (60.0%) |  |
|  | 2 | 12 (33.3%) | 8 (50.0%) | 4 (20.0%) |  |
|  | 3 | 6 (16.7%) | 2 (12.5%) | 4 (20.0%) |  |
|  | 4 | 1 (2.8%) | 1 (6.3%) | 0 (0.0%) |  |
| Regimen |  |  |  |  | 0.30 |
|  | Anti-PD-(L)1 monotherapy | 32 (88.9%) | 13 (81.3%) | 19 (95.0%) |  |
|  | Anti-PD-(L)1 + anti-CTLA-4 | 4 (11.1%) | 3 (18.8%) | 1 (5.0%) |  |
| Amplification of *ERBB2* |  |  |  |  | 1.00 |
|  | Amplification | 1 (2.8%)* | 1 (6.3%) | 0 (0.0%) |  |
|  | No amplification | 35 (97.2%) | 15 (93.8%) | 20 (100.0%) |  |
| EBV |  |  |  |  | 1.00 |
|  | Negative | 36 (100.0%) | 16 (100.0%) | 20 (100.0%) |  |
|  | Positive | 0 (0.0%) | 0 (0.0%) | 0 (0.0%) |  |
| PD-L1 (CPS) |  |  |  |  | 0.25 |
|  | <1 | 13 (36.1%) | 6 (37.5%) | 7 (35.0%) |  |
|  | ≥1 & <10 | 2 (5.6%) | 2 (12.5%) | 0 (0.0%) |  |
|  | ≥10 | 14 (38.9%) | 4 (25.0%) | 10 (50.0%) |  |
|  | NA | 7 (19.4%) | 4 (25.0%) | 3 (15.0%) |  |
| TMB |  |  |  |  | 0.005 |
|  | <40 | 9 (25.0%) | 10 (62.5%) | 1 (5.0%) |  |
|  | ≥40 | 27 (75.0%) | 6 (37.5%) | 19 (95.0%) |  |
| NGS testing |  |  |  |  | 0.73 |
|  | Plasma-based | 14 (38.9%) | 7 (43.8%) | 7 (35.0%) |  |
|  | Tissue-based | 22 (61.1%) | 9 (56.3%) | 13 (65.0%) |  |

Data are n (%) or median (IQR).

*The patient with amplified *ERBB2* gene was confirmed by 3+ HER2 IHC result.

Abbreviations: CPS=combined positive score, CTLA-4=cytotoxic T lymphocyte antigen 4, EBV=Epstein-Barr virus, ECOG=Eastern Cooperative Oncology Group, GEJ=gastroesophageal junction, HER2=human epidermal growth factor receptor 2, ICI=immune checkpoint inhibitor, IHC=immunohistochemistry, IQR=interquartile range, NGS=next-generation sequencing, NA=not applicable, PD-L1=programmed death-ligand 1.

## Supplementary Table S16. Univariable and multivariable analysis of PFS and OS in the ICI treatment cohort.

|  | Progression-free survival | | | | Overall survival | | | |
| --- | --- | --- | --- | --- | --- | --- | --- | --- |
|  | Univariable analysis | | Multivariable analysis | | Univariable analysis | | Multivariable analysis | |
| Parameter | HR (95%CI) | P value | HR (95%CI) | P value | HR (95%CI) | P value | HR (95%CI) | P value |
| Age (≥65 vs. <65) | 0.61 (0.23-1.62) | 0.32 |  |  | 0.71 (0.22-2.32) | 0.57 |  |  |
| Sex (male vs. female) | 0.89 (0.32-2.44) | 0.81 |  |  | 0.92 (0.27-3.16) | 0.89 |  |  |
| Primary location (GEJ vs. others) | 1.08 (0.25-4.75) | 0.92 |  |  | 0.86 (0.11-6.83) | 0.89 |  |  |
| Differentiation (moderate vs. poor/undifferentiated) | 0.55 (0.16-1.95) | 0.36 |  |  | 0.24 (0.03-1.88) | 0.18 |  |  |
| Stage (metastatic vs. locally advanced) | 3.57 (0.81-15.75) | 0.093 |  |  | 35.6 (0.16-8.0*10^3^) | 0.20 |  |  |
| ECOG (>0 vs. 0) | 0.71 (0.25-2.07) | 0.54 |  |  | 0.42 (0.13-1.38) | 0.15 |  |  |
| Treatment lines (≥2^nd^ vs. 1^st^) | 0.59 (0.22-1.58) | 0.29 |  |  | 0.61 (0.18-2.06) | 0.42 |  |  |
| PD-L1 |  |  |  |  |  |  |  |  |
| CPS≥1 vs. CPS<1 | 1.23 (0.37-4.04) | 0.74 |  |  | 1.32 (0.29-5.90) | 0.72 |  |  |
| CPS≥10 vs. CPS<10 | 1.31 (0.40-4.30) | 0.66 | 0.99 (0.29-3.35) | 0.99 | 1.96 (0.44-8.80) | 0.38 | 1.29 (0.28-6.05) | 0.75 |
| TMB |  |  |  |  |  |  |  |  |
| Continuous variable | 1.00 (0.99-1.01) | 0.55 |  |  | 1.00 (0.98-1.01) | 0.69 |  |  |
| Rank | 0.99 (0.94-1.04) | 0.65 |  |  | 1.00 (0.95-1.06) | 0.90 |  |  |
| ≥40 vs. <40 | 1.34 (0.43-4.20) | 0.61 | 0.48 (0.09-2.51) | 0.39 | 1.13 (0.30-4.26) | 0.86 | 0.34 (0.05-2.50) | 0.29 |
| NGS testing (tissue vs. plasma) | 0.96 (0.36-2.58) | 0.93 |  |  | 1.24 (0.68-2.24) | 0.49 |  |  |
| **Number of mutated members of PI3K-AKT-mTOR pathway (>1vs. 0/1)** | **3.40 (1.16-10.00)** | **0.026** | **5.99 (1.21-29.61)** | **0.028** | **3.59 (0.94-13.78)** | **0.063** | **11.88 (1.30-108.4)** | **0.028** |

Abbreviations: CI=confidence interval, CPS=combined positive score, ECOG=Eastern Cooperative Oncology Group, GEJ=gastroesophageal junction, HR=hazard ratio, ICI=immune checkpoint inhibitor, IHC=immunohistochemistry, NGS=next-generation sequencing, OS=overall survival, PD-L1=programmed cell death ligand 1, PFS=progression-free survival, TMB=tumor mutational burden.
